# On a Fluorescent Dye for Indicator Displacement From Cucurbit[7]uril‐Based Molecular Recognition: A Joint Experimental‐Computational Study

**DOI:** 10.1002/cphc.202500620

**Published:** 2026-03-31

**Authors:** Kevin Droguett, Angélica Fierro H., Mario Aranda, Costantino Zazza, Margarita E. Aliaga

**Affiliations:** ^1^ Departamento de Química Física Escuela de Química Facultad de Química y de Farmacia Pontificia Universidad Católica de Chile Santiago Chile; ^2^ Departamento de Química Orgánica Escuela de Química Facultad de Química y de Farmacia Pontificia Universidad Católica de Chile Santiago Chile; ^3^ Departamento de Farmacia Escuela de Química y Farmacia Facultad de Química y de Farmacia Pontificia Universidad Católica de Chile Santiago Chile; ^4^ Department for Innovation in Biological Agro‐food and Forest systems (DIBAF) Università degli Studi della Tuscia Viterbo Italy

**Keywords:** Cucurbit[7]uril, density functional theory, indicator displacement‐assay, quinolinone‐derivatives, supramolecular control

## Abstract

The host–guest complexation of a new fluorescent probe called 7‐(diethylamino)‐4‐hydroxyquinoline‐2(1H)‐one (**QD**) and its inclusion complex with the Cucurbit[7]uril (**CB7**) macrocycle were studied. **QD** was successfully synthesized, and an extensive characterization was performed for both the free probe and its corresponding **QD•CB7** adduct. Spectroscopic measurements indicate that the complexation enhances and promotes the photophysical properties of the **QD**, increasing its quantum yield and resulting in a 4.6‐fold increase in it. Aqueous **QD** and **QD•CB7** were also investigated, combining density functional theory wavefunctions with quantum theory of atoms in molecules descriptors and a semiclassical molecular dynamics technique. The derived picture suggests that the spontaneous **CB7**‐based molecular recognition mechanically hinders a light‐driven free rotation—along a diethylamino substituent within the **QD** molecule—avoiding the formation of a nonemissive torsional intramolecular charge transfer state. Moreover, the addition of Tyramine (**TA**), a biogenic primary amine with a high affinity for **CB7**, resulting in a fluorescence switch‐off response. These results demonstrate that the **TA** analyte effectively displaces the dye **QD** from the **CB7** host–guest complex. This dye is then proposed as a fluorescent indicator‐displacement assay for the detection and determination of the binding constants of biogenic amines.

## Graphical abstract

The host–guest complexation of a new fluorescent probe called 7‐(diethylamino)‐4‐hydroxyquinoline‐2(1H)‐one (QD) and its inclusion complex with the Cucurbit[7]uril (CB7) macrocycle were studied within a joint experimental and computational study. Confinement alters the photophysical properties of the synthesized quinolinone derivative also offering a potential fluorescent indicator‐displacement assay for the analytical estimation of the binding affinity of biogenic amines in aqueous solutions.

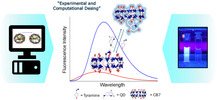

## 1 Introduction

It is known that small molecules can be included in synthetic macrocyclic receptors such as Calixarene, Cyclodextrin, Cucurbit[n]uril (CB[n]), among others. These macrocycles can modulate the properties of the guests [1, 2, 3] as their microenvironment changes [4, 5]. In particular, the CB[n] family of macrocycles has a symmetrical geometry with carbonylic portals and a hydrophobic cavity, with polarizability close to the gas phase condition [3, 6]. Specifically, cucurbit [7]uril (**CB7**) is broadly used thanks to its water solubility [7]. There is good evidence of the capability of this macrocycle to modulate the fluorescent quantum yield [8], pKa [9], fluorescent lifetime [10], solubility [11], and molar absorptivity coefficient [12]. Due to the rigid structures and efficient binding affinities, Cucurbit[n]urils combined with a variety of fluorescent guests have gained extensive utilization in indicator displacement assays (IDA) [13, 14, 15] for the determination of binding constants and stoichiometries of binding [16] and sensing non‐fluorescent or weakly fluorescent organic compounds (analytes) in a selective and specific manner [17]. In particular, novel fluorescent‐indicator displacement assays (F‐IDAs) utilizing **CB7** as a supramolecular host have been recently reported in literature [18, 19, 20, 21].

Due to their properties, quinolinone derivatives are attractive molecules for the development of optical probes. A variety of examples with this core have been reported as antifungal, antibiotic, and optical sensors [22, 23, 24, 25, 26]. Substitution on the 7‐position of quinolinone derivatives promotes electron delocalization due to the push‐pull [27] effect, thus improving their optical properties. Amino‐containing substituents are often used thanks to their donor capability, promoting a good intramolecular charge transfer (ICT) state [28, 29, 30]. Additionally, the free rotation of this substituent can generate a torsional intramolecular charge transfer (TICT) state [31], which is generally nonemissive. Thus, the presence of a nonemissive state is attractive for potential fluorescent probes [31], as cucurbituril macrocycles have proven their capability to inhibit such torsion [32, 33, 34].

There are several molecules worthy of being studied through an IDA process; in this context, it is well known that biogenic amines represent another important class of target analytes due to their physiological activity [35, 36]. In particular, tyramine (**TA**) is found in different food matrices [37, 38], and its overconsumption leads to several health risks making this biogenic amine a good analyte to study through the incorporation of chromogenic or fluorogenic agents. Studies on the molecular recognition of biogenic amines by cucurbiturils have shown great promise for estimating the binding free energies (Δ*G*) of encapsulation of monoamine neurotransmitters [39] (and quoted references therein cited.) Because of their exceptional recognition properties in aqueous medium, cucurbiturils are continuously generating a tremendous interest in the supramolecular community [40].

**FIGURE 1:**
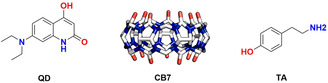
Chemical structures of 7‐(diethylamino)‐4‐hydroxyquinoline‐2(1H)‐one dye **QD**, the macrocycle cucurbit [7]uril **CB7** and Tyramine (**TA**).

Taking all of this into consideration, we aimed to develop an experimental and theoretical study of a supramolecular system formed with a 7‐(diethylamino)‐4‐hydroxyquinoline‐2(1H)‐one derivative (**QD**) as a guest molecule and the **CB7** macrocycle as a host, see Figure 1. We combined in a self‐consistent way experimental measurements and simulations, essentially based on converged electronic wavefunctions within density functional theory (DFT) [41] framework and all‐atom classical molecular dynamics propagators [42], to fully analyze the electronic and structural properties of the **QD** molecule and the **QD•CB7** supramolecular complex. The derived picture suggests that a spontaneous **CB7**‐based molecular recognition mechanically hinders a light‐driven free rotation—along a diethylamino substituent within the **QD** molecule—avoiding the formation of a nonemissive TICT state. On the contrary, we observe that the addition of **TA** results in a fluorescence switch‐off response due to a competitive displacement of the guest species.

## 2 Experimental Methods

### 2.1 Reagents, Materials, and Synthesis

All reagents and materials were purchased from Sigma–Aldrich. **CB7** solution was prepared with ultrapure Mili‐Q water, the purity and concentration were determined by titration with cobaltocenium cation using UV–vis spectroscopy [43].

**QD** synthesis using a mixture of ethyl malonate (3.6 mmol, 0.55 mL) and 3‐amino‐N, N‐diethylaniline (3.0 mmol, 500 mg) with 1 mL of HCl 25% v/v was stirred at 130°C overnight. Then, the round‐bottom flask is cooled at room temperature, and the result is poured into a saturated sodium carbonate solution. Finally, the product is extracted and purified by thin layer chromatography (TLC) with chloroform:methanol (9:1) as eluent, giving a pale pink powder of 7‐(diethylamino)‐4‐hydroxyquinolin‐2(1H)‐one (**QD**) (254 mg, 1.1 mmol, 36% yield) ^1^H NMR (400 MHz, DMSO‐d6) δ 10.78 (s, 1H), 10.59 (s, 1H), 7.51 (d, J = 9.0 Hz, 1H), 6.54 (dd, J = 9, 2.4 Hz 1H), 6.42 (d, J = 2.4 Hz 1H), 5.39 (s, 1H), 3.35–3.5 (q, 4H), 1.11 (t, J = 6.9 Hz, 6H). ^13^C NMR (101 MHz, DMSO‐d6) δ 164.70, 163.82, 149.79, 141.85, 124.26, 107.46, 105.24, 95.11, 94.07, 44.34, 12.92. [M+H]^+^ calculated for C_13_H_18_N_2_O_2_ 233.12, found m/z 233.05.

### 2.2 NMR and Mass Spectrometry Experimental Conditions

A Bruker Avance 400 MHz spectrometer was used to obtain ^1^H and ^13^C NMR spectra at 298 K with dimethylsulfoxide‐*d*6 (DMSO‐*d*6) solvent. The spectra were produced with MestreNova v14.2 software.

The obtained product was also analyzed using a Waters (Milford, MA, USA) Arc HPLC system composed of a Quaternary Solvent Manager‐R pump, Sample Manager FTN‐R autosampler, CH‐30A column heater, and UV detector coupled to Acquity QDa mass spectrometer equipped with an electrospray ionization (EI).

Separation of **QD** was carried out on Phenomenex (Torrance, CA, USA) Kinetex 3.5 µm XB‐C18 column (150 × 4.6 mm, 100 Å) set at 30°C, using a binary mobile phase composed of acidified water (0.1% v/v formic acid‐A) and acidified acetonitrile (0.1% v/v formic acid‐B). The following gradient program was applied at a flow rate of 0.5 mL min^−1^: 0–5 min 60%–40% B; 5–7 min 10%–90% B; 7–8 min 50%–50% B; 9–15 min 60%–40% B (column conditioning) for a total run time of 15 min.

UV‐detection was performed at 350 nm, and MS analysis was carried out using the following settings: EI (‐) capillary voltage of 0.8 kV, cone voltage of 15 V, probe temperature of 600°C, and source temperature of 120°C. Full Scan mode (m/z 100–1000) was used for qualitative analysis.

High‐Resolution (HR) QTOF‐MS Studies. Mass spectrometry (HR‐MS) experiments were conducted using a compact QTOF (Bruker) with an ionization voltage of 4 kV and negative polarity. The scan parameters were as follows: mass range: 50−1300 m/z, spectra rate: 5 Hz, nebulizer: 0.6 bar, dry gas: 5 L/min, dry temp: 200°C.

### 2.3 Spectroscopic Experiments

A Cary 60 spectrometer from Agilent Technologies was used for UV–vis experiments. The emission was measured using a Fluorescent Spectrometer, Horiba FluoroMax‐4. Both were recorded using quartz cuvettes with 1 cm of optic path at *T* = 298.0 ± 0.1 K.

Time‐resolved fluorescence was measured using a Lifespec II picosecond fluorescence lifetime spectrometer Edinburgh Instrument. With a 380 nm laser diode as an excitation source, a fast red‐sensitive PMT detector and 10,000 maximum counts were collected for the determination of fluorescence lifetime at *λ*_max_. Instrument response function (IRF) was measured with a diluted Ludox solution. The fluorescence lifetime was obtained with a deconvolution of the IRF, and the fluorescence decay [44].

The fluorescent quantum yield was determined using the methodology reported in our previous report. We use quinine sulfate in 0.5 M H_2_SO_4_ as a standard ($\phi_{s}$ = 0.55 at 380 nm) [45].

The binding affinity (*K*_b_), which quantifies the strength of interaction between a generic ligand and its receptor at nanoscale level, was obtained using a 1:1 fitting model by emission spectroscopy using the equation [46, 47]

(1) $${\left[HG\right]}_{Eq}^{2}-\left({\left[H\right]}_{T}+{\left[G\right]}_{T}+\frac{1}{Ks}\right){\left[HG\right]}_{Eq}+{\left[H\right]}_{T}{\left[G\right]}_{T}=0$$

where *H* is the host (**CB7**), *G* is the guest (**QD**), and *HG* is the complex. *K*_b_ was then calculated according to previous reports [47].

Thermodynamical experiments were conducted using a PEAQ‐ITC Malvern Panalytical. The titration of **QD** using **CB7** was measured in water: dimethylsulfoxide (99:1) solution at 298 K. The result was processed using the PEAQ‐ITC Analysis Software. Finally, indicator displacement assay (IDA) was conducted by fluorescence spectroscopy using a competitive model according to the equation

(2) $$K_{2}^{2}{\left[HI\right]}_{Eq}^{3}+(2K_{2}+K_{1}-K_{2}K_{1}{\left[H\right]}_{T}-3K_{2}K_{1}{\left[I\right]}_{T}){\left[HI\right]}_{Eq}^{2}+(K_{2}K_{1}{\left[H\right]}_{T}{\left[I\right]}_{T}-K_{1}{\left[H\right]}_{T}-K_{s1}{\left[I\right]}_{T}-K_{2}K_{1}{\left[I\right]}_{T}^{2}-1){\left[HI\right]}_{Eq}+K_{1}{\left[H\right]}_{T}{\left[I\right]}_{T}=0$$

*K*_1_ and *K*_2_ are the binding constants for the indicator (**QD**) and the guest (**TA**) toward the host (**CB7**), respectively.

### 2.4 Computational Details

**QD** molecule was optimized in a DFT scheme [41] adopting the hybrid exchange‐correlation Becke 3‐parameters Lee–Yang–Parr (B3LYP) functional in conjunction with the 6‐311++G** atomic basis set [48, 49, 50, 51]. Also, we used Grimme's dispersion correction as an add‐on to standard Kohn–Sham (KS) equations with the original D3 damping function (i.e., DFT‐D3) [52]. This molecule was primarily relaxed by applying a redundant‐based internal coordinates algorithm in ideal gas‐phase condition, and, subsequently in solution mimicking the dilute water solution via the popular Conductor‐like Polarizable Continuum Model (C‐PCM) model [53]. Time‐Dependent (TD‐DFT) computations are applied to estimate the lowest‐lying electronic excitations of **QD** in a water medium [54]. Always remaining in this field, we computed the intramolecular charge transfer process accompanying the transition toward the first singlet excited state (i.e., from GS to S_1_) considering both the optimized ground state and excited state, at C‐PCM(H_2_O)/B3LYP(D3)/6‐311++G** level, while splitting up the **QD** molecule in two fragments: the —N(CH_2_CH_3_)_2_ substituent and the remaining part of the molecular structure. The interfragment charge transfer during the excitation is estimated via IFCT (InterFragment Charge Transfer) theory with a Becke partitioning incorporated in the Multiwfn 3.8 program [55, 56]. Relaxed Potential Energy Surface Scans (PESs) were also computed along a torsional‐like motion of the —N(CH_2_CH_3_)_2_ moiety with respect to the aromatic counterpart. The Gaussian 16 code (Rev. C01) was adopted for the DFT computations [57]. The obtained **QD** structure was then used to generate a classical all‐atoms atomic force field; bond, angle, dihedral, and Lennard‐Jones OPLS‐AA parameters were extracted from LigParGen web‐based service offered by the Jorgensen group [58]; initial charges were estimated using the Molecular Electrostatic Potential (MEP) method of Merz–Kollman [59], and all bonds involving hydrogen atoms were constrained using the LINCS algorithm [60]. As a next step, the solvation dynamics of aqueous **QD** was analyzed carrying out a 200 ns MD simulations at 298 K in an NVT ensemble considering this solute at the center of a cubic box with an edge of 44 Å; solvent molecules were treated according to the SPC scheme [61] and MD simulations were performed using GROMACS Software (release 2022.6) [62]. Furthermore, with the target of theoretically modeling—from a dynamical point of view—the lowest valence **QD** UV–vis absorption bands herein experimentally detected, we used a TD approach over an adaptive QM/MM/C‐PCM partitioning scheme as already proposed over similar systems by one of us (CZ) [63, 64, 65]. The UV–vis absorption spectrum is then estimated by combining vertical excitation energies with oscillator strengths and using Gaussian broadening functions having a full width at half maximum (FWHM) of 3000 cm^−1^ (i.e., the Δ*v′*_1/2_) [66]. The formula used to convolute the computed absorption spectra is given below

(3) $$\varepsilon \left(v\prime \right)=\frac{2.175\times 10^{8}}{\text{Δ}v_{\frac{1}{2}}^{\prime }}\times f\times \exp\left[-2.772\times \frac{{\left(v\prime -v_{i\to f}^{\prime }\right)}^{2}}{\text{Δ}v_{\frac{1}{2}}^{\prime }}\right]$$

where *f* (the oscillator strength under the transition dipole length approximation) and *v′*_*i→f*_ (the vertical excitation energy in wavenumbers, cm^−1^) are both extracted from quantum mechanical calculations at TD‐DFT level of theory. The oscillator strength is related to the transition dipole moment, *M*_*i→f*_ by the standard equation

(4) $$f_{i\to f}=\frac{8\pi m_{e}}{3e^{2}h^{2}}\times \text{Δ}E_{i\to f}\times {\left|M_{i\to f}\right|}^{2}=\frac{8\pi m_{e}c}{3e^{2}h^{2}}\text{×}v_{i\to f}^{\prime }\times {\left|M_{i\to f}\right|}^{2}$$

where *m_e_* and *e* are the mass and the charge of the electron, respectively, *h* is Planck's constant, and *c* is the speed of light. The energy difference (Δ*E*_*i→* *f*_) between these states is related to the frequency *v*, the wavelength *λ* and the wavenumber *v′* of absorbed light by the equation

(5) $$\text{Δ}E_{i\to f}=h\times v_{i\to f}=h\times \frac{c}{\lambda_{i\to f}}=h\times c\times v_{i\to f}^{\prime }$$

**FIGURE 2:**
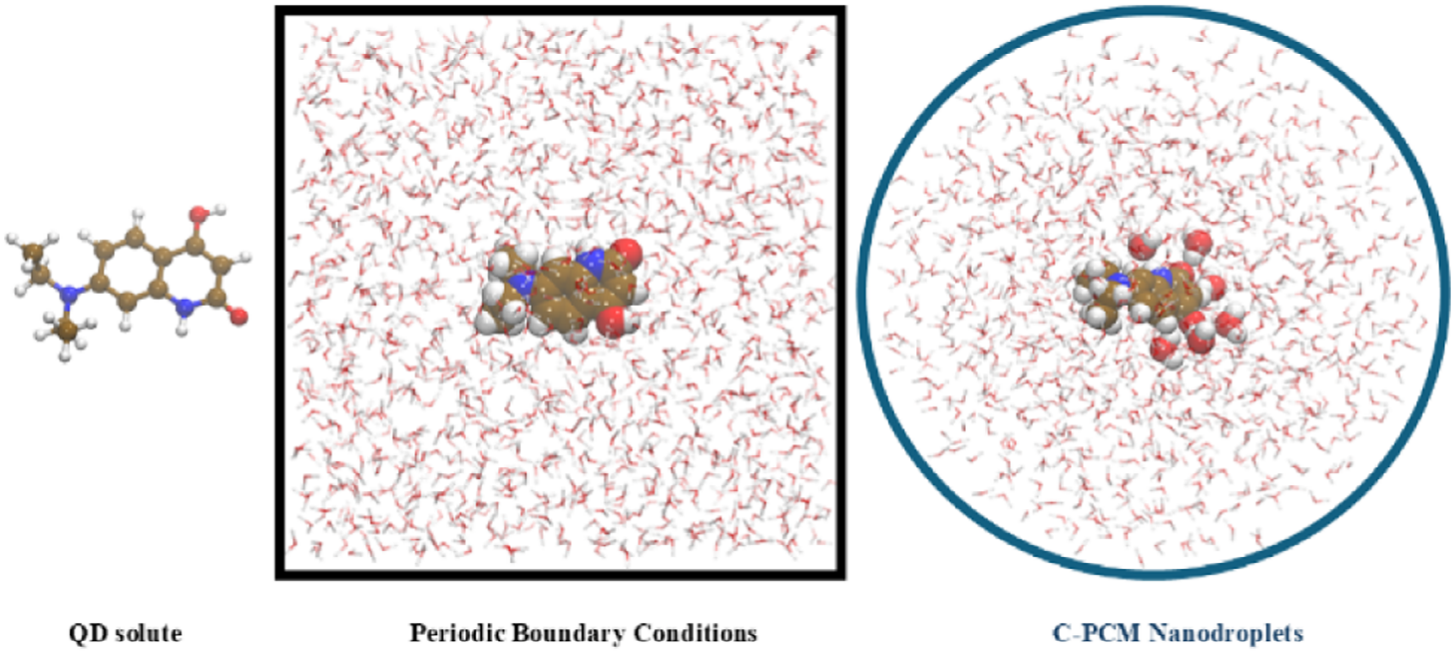
From left to right: the investigated **QD** solute, the applied classical simulation cubic box with aqueous **QD**, the C‐PCM spherical nanodroplets extracted from the equilibrated MD trajectory at 298 K hosting an adaptive QM/MM modeling.

The results of a TD‐DFT calculation are then convoluted using Gaussian‐type curves to create UV–vis spectra; more specifically, each electronic transition is associated with a Gaussian curve accordingly to the expression of the *ε*(*v′*) value, which provides an estimation of the molar absorption coefficient, in M^−1^ cm^−1^, as reported in Equation (3). In practice, we considered a spherical cavity with a radius of 20 Å with the origin centered over the geometrical center of the **QD** solute treated at B3LYP(D3) level (see Figure 2), and we extracted from the MD trajectory of 200 ns a snapshot every 10.0 ns. The encircling solvent molecules were considered within the perturbing classical (MM) region only in cases in which their COM distance was shorter than the imposed spherical cut‐off. Such a solute results then effectively confined within a subset of different spherical nanodroplets in interaction with solvent treated both by explicit molecules (treated at QM level or as atomic point charges) and implicit ones (*i.e.*, a spherical C‐PCM cavity, see Figure 2). In this respect, a subset of water molecules featuring H‐bonding contacts with the QD molecule was treated at QM level within the proposed QM/MM/C‐PCM approach. These interactions are actually based on two imposed cutoffs: i) the Acceptor–Donor–Hydrogen (Ac–Dn–H) angle between the three atoms involved in the H‐bond; j)the distance between the two heavy atoms involved in the interaction (Ac–Dn); the criteria we selected are as follows: Ac–Dn–H angle ≤ 30° and Ac–Dn distance ≤ 3.5 Å [63, 64].

Subsequently, we focused our attention on the nature of the supramolecular interactions, finely modulating the **QD•CB7** supramolecular assembly in a water environment with a topological description within the quantum of theory of atoms in molecules (QTAIM) [67, 68, 69, 70, 71]. The derived DFT‐based indicators, at C‐PCM/B3LYP(D3)/6‐311++G** level of theory, mainly include the Kohn–Sham (K‐S) electron density *ρ*(***r***) [41], the electronic energy density *H*(***r***) and its kinetic and potential components *G*(***r***) and *V*(***r***), respectively and the Reduced Density Gradient (RDG) *s*(***r***). The *ρ*(***r***) is defined by the equation

(6) $$\rho \left(r\right)=\sum_{i}\eta_{i}{\left|\varphi_{i}\left(r\right)\right|}^{2}$$

where $\eta_{i}$ is the occupation number of the natural orbital $\varphi_{i}$, in turn expanded as a linear combination of the atomic basis functions [67, 68, 69, 70, 71]. The *H*(***r***) is the sum of the kinetic energy density *G*(***r***) and the potential energy density *V*(***r***)

(7) $$H\left(r\right)=G\left(r\right)+V\left(r\right)$$

The presently‐employed definition of the *G*(***r***) is given by the equation

(8) $$G\left(r\right)=\frac{1}{2}\sum_{i=1}\eta_{i}{\left|\nabla \varphi_{i}\left(r\right)\right|}^{2}$$

with the sum running over all the occupied natural orbitals $\varphi_{i}$ of occupation numbers $\eta_{i}$. The potential energy density *V*(***r***) is evaluated [67, 68, 69, 70, 71] from the local form of the virial theorem

(9) $$V\left(r\right)=\frac{1}{4}\nabla^{2}\rho \left(r\right)-2G\left(r\right)$$

The RDG—coming from the *K*–*S* electronic density and its first derivative—is defined by the following equation [72, 73]

(10) $$s\left(r\right)=\frac{\left|\nabla \rho (r)\right|}{2{\left(3\pi^{2}\right)}^{\frac{1}{3}}\times \rho {\left(r\right)}^{\frac{4}{3}}}$$

**FIGURE 3:**
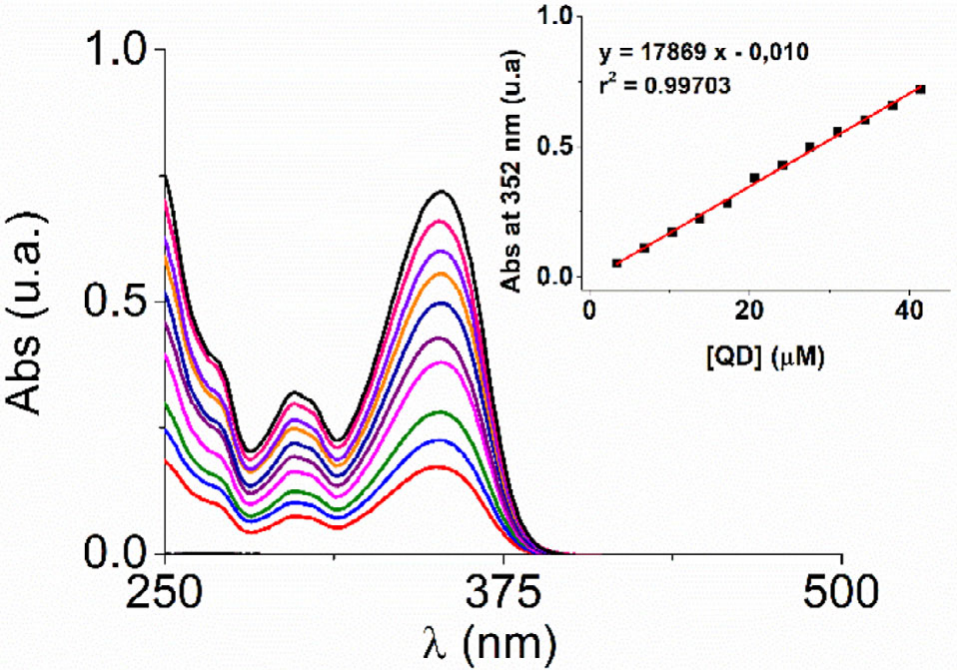
UV–vis absorption spectra of **QD** (3–40 μM) in water/DMSO mixture (99/1) at 298 K. Inset: plot of absorbance at 352 nm *vs* concentration of **QD**.

Low‐value reduced density gradient (RDG) isosurfaces, often used in analyzing non‐covalent interactions (NCIs), are characterized by relatively low values of *s*(***r***), typically between 0.3 and 0.6 [73, 74, 75, 76, 77, 78]. These isosurfaces appear when there are interacting atoms, and they are particularly associated with regions of low electron density—typically at around 0.05 *e*·*a*_0_^−3^—where NCIs occur. The low‐*s*(*r*)*/low‐ρ*(*r*) isosurfaces are, in turn, mapped in terms of the *sign*(*λ*_2_) *× ρ*(*r*), *λ*_2_ being the second eigenvalue (*λ*_1_ *< λ*_2_ *< λ*_3_) of the Hessian matrix of *ρ*(***r***)*.* In essence, the sign of *λ*_2_ is used to distinguish between attractive (*λ*_2_ < 0) and repulsive (*λ*_2_ > 0) interactions, and the value of *ρ*(***r***) is exploited to rank the corresponding strength [73]. Thus, the NCIs are conveniently visualized considering the optimized structure, at C‐PCM/B3LYP(D3)/6‐311++G** level of computation, of the investigated **QD•CB7** supramolecular assembly in a 3D space by plotting a chosen low‐value isosurface of the *s*(***r***), colored by the *sign*(*λ*_2_) × *ρ*(*r*) (standard employed colors range from blue to red for attractive to repulsive interactions, respectively); also this analysis was carried out with the Multiwfn program [55, 56]. The 3D plots of the *s*(***r***) *vs sign*(*λ*_2_) *× ρ*(*r*) were realized with the Visual Molecular Dynamics (VMD) program [79] setting *sign*(*λ*_2_) *× ρ*(*r*) between −0.050 *e·a*_0_^−3^ (blue) and 0.010 *e·a*_0_^−3^ (red). At last, Hirshfeld surface analysis [80] was also applied to further visualize and quantify intermolecular interactions within **QD•CB7** system since it helps understand how these molecules interact, revealing important details about noncovalent interactions like hydrogen bonds and π–π stacking interactions. (In this respect, it is worth noting that: (i) we considered, within the applied TD‐DFT framework, only the first thirty singlet excitations since we are focused on the interval depicted in Figure 3; (ii) the Cam‐B3LYP [81] functional (data not shown) provided a very similar excitation pathway with wavelengths blue‐shifted by almost 10 nm if compared with the data collected in Table S1).

## 3 Results and Discussion

### 3.1 Characterization of Quinoline‐2(1H)‐One Derivative and QD Absorptions

**FIGURE 4:**
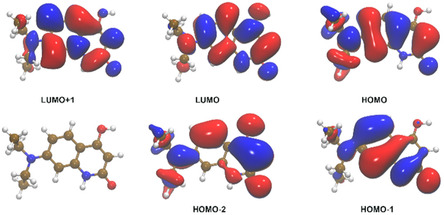
**QD** *K*–*S* molecular orbitals—as extracted at C‐PCM(H_2_O)/B3LYP(D3)/6‐311++G** level—involved in the electronic excitations collected in Table S1a featuring oscillator strengths > 0.15 a.u.; the blue color reflects the positive (+) counterpart of the corresponding eigenvector and an isodensity surface plot value of 0.015 *e*·*a*_0_^−3^ is represented. The associated eigenvalues (in eV) are: −7.10, −6.47, −5.54 (HOMO), −1.40 (LUMO), −0.46.

**SCHEME 1:**
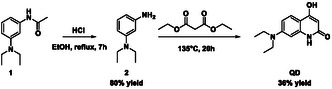
Synthesis of **QD** molecular system with the associated yield.

The synthesis of this quinolinone derivative was carried out by a Knorr quinoline synthesis starting with the aniline (**2**) (previously obtained by hydrolyzation, see Scheme 1). The probe was extensively characterized by NMR in Figure S1–S5, and mass spectrometric analysis is shown in Figure S6. It is important to note that the ^1^H ppm shifts correspond to the lactam form of the quinolinone, which was corroborated with Heteronuclear Single Quantum Coherence (HSQC) and Heteronuclear Multiple Bond Correlation (HMBC) techniques. The UV–vis absorption spectra of increasing concentration values of the **QD** in water/DMSO mixture (99/1) are depicted in Figure 3. The revealed absorbances between 240–500 nm indicate the presence of a well‐defined transition lying at around 352 nm with a molar absorptivity coefficient of 1.79 × 10^4 ^ M^−1^ cm^−1^ with a second band centered at 300 nm featuring a lesser intensity if compared with the lowest‐energy signal. The experimental measurements also report a subset of absorptions whose summation generates a third absorption profile at excitation wavelengths lower than 270 nm. In addition, the UV–vis spectra acquired in the laboratory have also been also deeply analyzed combining classical molecular simulations of aqueous **QD** with an adaptive QM/MM/C‐PCM scheme (see Subsection 2.4). In this respect, it should be noted that before simulating the UV–vis spectra of the **QD** solute with a mixed QM + MM algorithm we have estimated the low‐lying singlet excitation transitions with the implicit C‐PCM solvation model, and the results are collected in Table S1a and Figure 4. Looking at these data it is easy to realize that the first UV–vis absorption band essentially presents a HOMO(H) → LUMO(L) single excitation contribution (i.e., 95%) reflecting a valence ^1^π–π* character. Furthermore, the derived absorption maximum found at 334 nm (*f* = 0.47) results only slightly underestimated with respect to the experimental value falling at ≈350 nm (see Figure 3). The remaining part of the absorption spectra still presents excitation pathways strongly modulated by ^1^π–π* transitions arising from H‐1‐>L (52%), H‐>L + 1 (41%) excitations [in the case of the absorption band at 270 nm (*f* = 0.18)] and from H‐1‐>L + 1 (67%), H‐1‐>L + 2 (16%) excitations [when the largest absorption at 221 nm (*f* = 0.65) is concerned].

**FIGURE 5:**
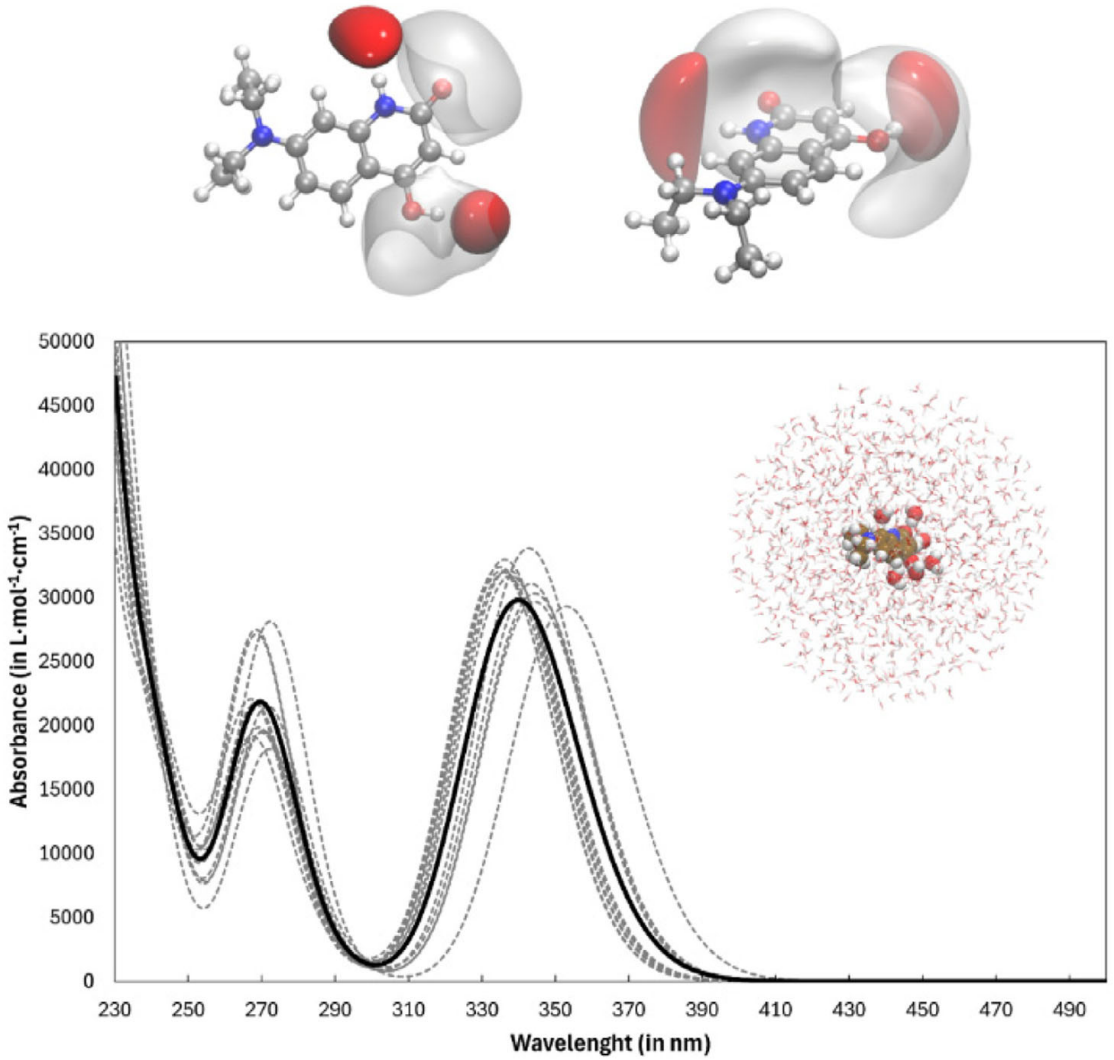
**QD** UV–vis absorption spectra—black solid line at [B3LYP(D3)/6‐311++G**/MM + C‐PCM] level—in H_2_O at 298 K. We considered 10 equally spaced MD (200 ns at 298 K, gray dashed lines) frames, and the excitations are computed using spherical nanodroplets. In the upper panel we show the water density regions (O in red, H in light gray) featuring solvent molecules in H‐bond contact with the investigated solute.

The absorption bands of **QD** are subsequently estimated via QM[B3LYP(D3)]/6‐311++G**)/MM/C‐PCM decomposition, and the convoluted spectra that have emerged are displayed in Figure 5. Such a UV–vis absorption spectrum actually features a shape in line with the experimental observations with: (i) a single peak profile with a *λ*_max_ lying at 341 nm (*λ*_exp _ ≈ 352 nm, see Figure 3) clearly undergoing a redshift by 7 nm toward the experimental maximum with respect to the value estimated at C‐PCM/B3LYP level; (ii) a second absorption at 270 nm resulting slightly underestimated by 20 nm (*λ*_exp _ ≈ 290 nm; (iii) a well pronounced absorption at *λ*_max_ < 240 nm. Moreover, we observe that nanosolvation patterns driven by thermal fluctuations and noncovalent interactions finely modulate the spectroscopic properties of **QD** in water solution. The lowest ^1^π–π* energy band estimated at 341 nm—QM[B3LYP(D3)]/6‐311++G**)/MM/C‐PCM level—features excitation wavelengths spanning a spectral region from 336 to 343 nm with an average value of 340 nm and an associated standard deviation (*σ*) of 6 nm. The corresponding oscillator strengths result as well tuned by solvation effects, spanning values between 0.40 and 0.47. A similar modulation, albeit to a lesser extent, has been also observed in the excitation band at *λ*_max _ ≈ 270 nm; the derived excitation wavelengths result confined in the range 268–275 nm (*σ* = 2 nm) with *f* between 0.24 and 0.35. The moderate shifts observed in these electronic transitions upon addition of explicit solvent molecules and thermal effects are essentially due to the ^1^π–π* character of the most intense excitations that are not accompanied by a drastic rearrangement of the electronic density during the absorption of light. At last, according to the imposed geometrical parameters (cutoff angle ≤ 30° and distance ≤ 3.5 Å) QM‐based solvation effects are only ascribed to H‐bond contacts between water molecules and the N—H, C—O and O—H functional groups of the **QD** solute. To better highlight this aspect, water density maps around the **QD** are calculated using the positions of either oxygen or hydrogen atoms of the water molecules along the MD trajectory of 200 ns at 298 K; this analysis reveals the presence of well‐structured first hydration layers encircling the amine‐hydrogen, the carbonyl oxygen and the opposite hydroxyl substituent. Some peculiar features have been pointed out by inspection of the radial distribution functions (RDFs). The solvent distribution around the amine‐hydrogen atom shows a first solvation shell with a maximum [*R*_max_(N—H‐‐O_W_)] at 1.98 Å and an extension up to 2.62 Å (see Figure S7). Moreover, the coordination number arising from the numerical integration of the first main peak of the RDF suggests the presence of a single solvent molecule in electrostatic interaction with the amine‐hydrogen. Around the proximal carbonyl oxygen, the RDF highlights the presence of a first prominent water layer peaked at 1.96 Å [*R*_max_(C—O‐‐H_W1, W2_)] with a minimum at almost 2.7 Å. The integral up to the minimum value of the first peak in the RDF estimates—on average—a coordination number suggesting the presence of ~two simultaneous water molecules at H‐bond distances from the carbonyl oxygen atom (Figure S7). As far as the hydroxyl group of **QD** is concerned, numerical values indicate the most statistically recurrent O—H‐‐O_W_ distances to be equal to 1.62 Å. Such a prominent peak (with a minimum at 2.34 Å) remarks the presence of a well‐defined solvent solvation shell encircling the O—H group of the **QD** solute (see Figure 5, upper panel).

### 3.2 Characterization of a TICT‐Modulated Process in H_2_O

As a next step, we focused on the intramolecular charge transfer process in aqueous **QD** molecule. We estimated via IFCT methodology the flow of electronic charge accompanying GS → S_1_(H → L) transition, dividing such a molecule into two fragments: fragment 1 is composed of the **QD** molecule minus the —N(CH_2_CH_3_)_2_ substituent, fragment 2 is the —N(CH_2_CH_3_)_2_ substituent as well. The IFCT methodology counts the electron and hole contributions from both fragment 1 and fragment 2. The difference between the number of electrons and holes in fragment 1 corresponds to the number of charges transferred from fragment 1 to fragment 2, whose ratio is defined as the Charge Transfer (CT) ratio. Meanwhile, the ratio of charges localized within the same fragment is treated as the Local Excitation (LE) ratio [55, 56]. Essentially, a higher LE% indicates that the excitation is primarily confined within a specific region of the molecule, while a higher CT% suggests that it involves a transfer of charge between different parts of the molecule itself. When the optimized molecular geometry of the GS is concerned, we can appreciate an intramolecular CT process of 0.281*e* from mainly the nitrogen lone pair of the —N(CH_2_CH_3_)_2_ substituent (fragment 2) to the remaining part of the **QD** molecule labeled as fragment 1. The CT percentage was found to be 28.1% while the LE was at 71.9%. In Figure S8a, we report the electron–hole distribution of the first electronically excited state of aqueous **QD** at the ground state geometry.

**FIGURE 6:**
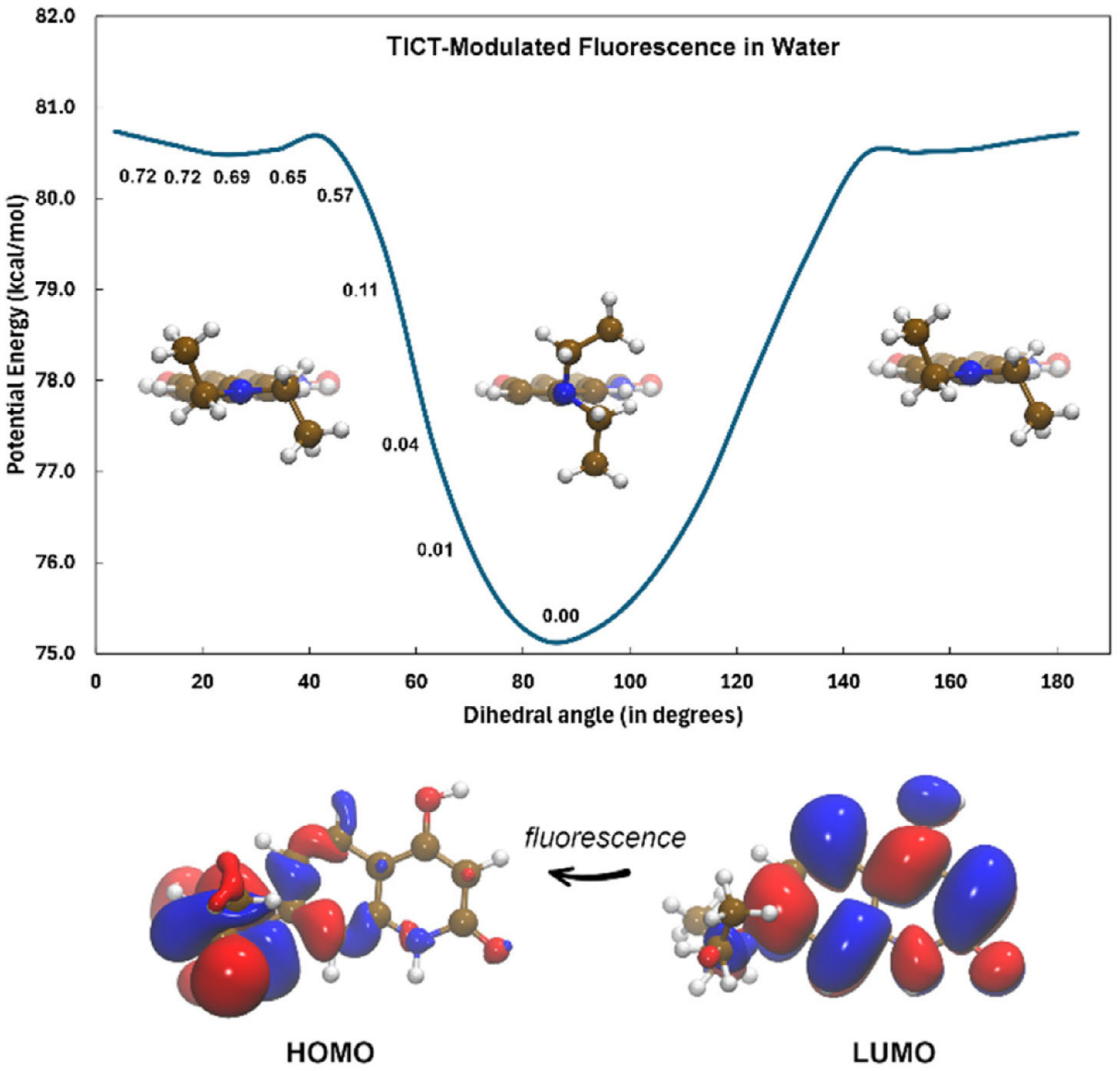
Relaxed PES computed considering the aqueous **QD** in the singlet (*S*_1_) electronic excited state at C‐PCM/B3LYP(D3)/6‐311++G** level. In the singlet excited state profile, we also report the associated oscillator strength (*f*) within Franck–Condon approximation during fluorescence emission. Representative geometries are also displayed, as well as the *K*–*S* molecular orbitals characterizing the observed quenching in the fluorescence emission within the singlet conformational basin.

The same IFCT analysis carried out upon having optimized the geometry of the first excited state reveals that, during the fluorescence process, a similar amount of electronic charge (0.321*e*) returns to fragment 2 (see Figure S8b); the CT% is found lying at 32.1, while the LE% counterparts at 67.1. Another interesting thing to mention is that the first electronic excited state, when relaxed, presents a limited (almost 20°) torsional‐like motion of the —N(CH_2_CH_3_)_2_ substituent along the C(fragment 1)–N(fragment 2) covalent axle. On the other hand, magnifying the observed rotational motion with a relaxed PESs scan of 180° we were able to appreciate a further and more pronounced conformational basin reflecting an electronic excited state with a different *K*–*S* orbitals decomposition, see Figure 6. Another interesting aspect to mention is the mismatch of the TD‐DFT oscillator strength (*f*) in the two sampled domains. In the case in which the fluorescence is estimated to occur in a “vertical subspace”, the return to the electronic **QD** ground state should be plausibly characterized by bright emission lines in the visible spectrum (*f * ≈ 0.7, see upper panel in Figure 6). Instead, a quenching of this signal (*f * ≈ 0.0) is expected as the **QD** exits from the aforementioned local basin and then presents itself in a cross‐shaped structure; this is basically because such a relaxation process removes the previously observed ^1^π–π* character of the emission process involving HOMO and LUMO orbitals depicted in Figure 4. This substantially non‐emissive (and not vertical) excited state results characterized—during the fluorescence process—by an inter‐fragment CT process of 0.825*e* from fragment 2 → fragment 1; the CT% is found at 83.6 and the LE% counterpart at 16.4. Considering these findings, it is evident that **QD** chromophore might result in particularly attractive IDA species within confined local environments. Such a computationally estimated TICT‐Modulated change in fluorescence is then subsequently used in this research to detect the host(**CB7**)–guest(**QD**) self‐assembly in water (see subsection 3.3). Finally, we would like to mention that the most stable structure of the **QD** system displays the two dimethyl groups in the opposite position (see Figure 6). The energy difference with respect to the conformation with the dimethyl groups on the same side results confined within 0.49 kcal/mol. Moreover, the electronic excitations result substantially unaltered (see Table S1b).

### 3.3 Host(CB7)–Guest(QD) Self‐Assembly in Water

**FIGURE 7:**
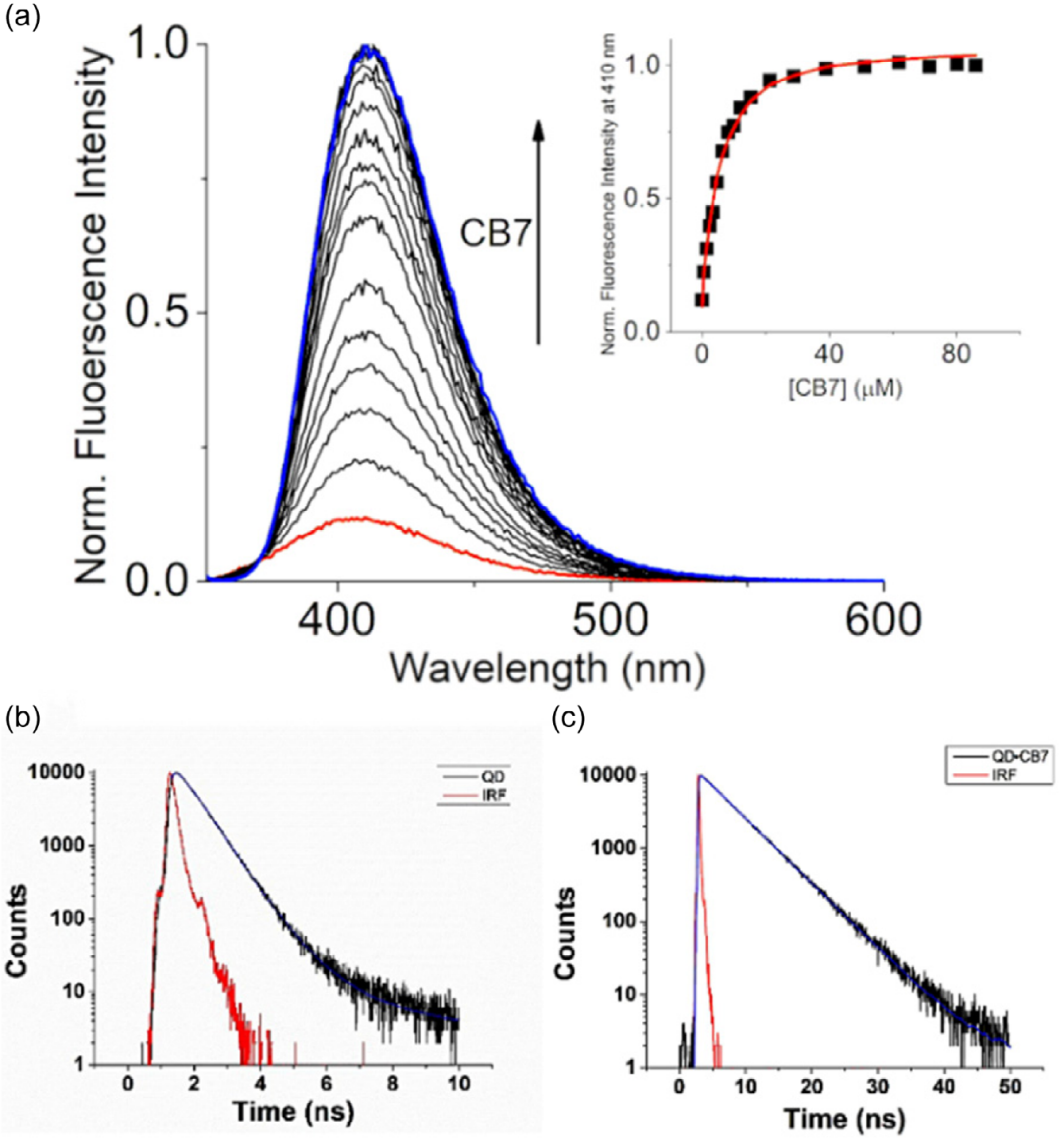
(a) Fluorescence Intensity dependence of **QD** (1.16 μM) after adding **CB7** (0–90 μM). The insert shows the fit for a complex model of 1:1 stoichiometry (K: 4.54 × 10^5^ ± 0.58 M^−1^). The emission was recorded exciting at 350 nm with a 1 nm slit at 298 K. Time fluorescent profile of (b) **QD** (1 μM) and (c) **QD** (1 μM) in the presence of **CB7** (60 μM). The excitation source was a near‐ultraviolet laser diode of 375 nm. Instrumental response function (IRF).

We studied the formation of supramolecular complexes through fluorescent means. Figure 7 shows the analytical variation of **QD** fluorescence intensity after the addition of increasing concentrations of **CB7** macrocycle. As can be seen, the intensity of **QD** in water reveals a clear hyperchromic shift upon the addition of such a macrocycle (in a 1:1 ratio). This spectroscopic variation, when associated with a self‐assembly of a host(**CB7**)–guest(**QD**) system, allows us to determine the associated binding constant using Equation (1), which results in a value of 4.54 × 10^5^ M^−1^. The nature of the observed hyperchromic shift was explored by studying the quantum yield ($\phi_{f}$) and fluorescent lifetime (***t***_F_) of the free probe and the **QD•CB7** supramolecular adduct.

**FIGURE 8:**
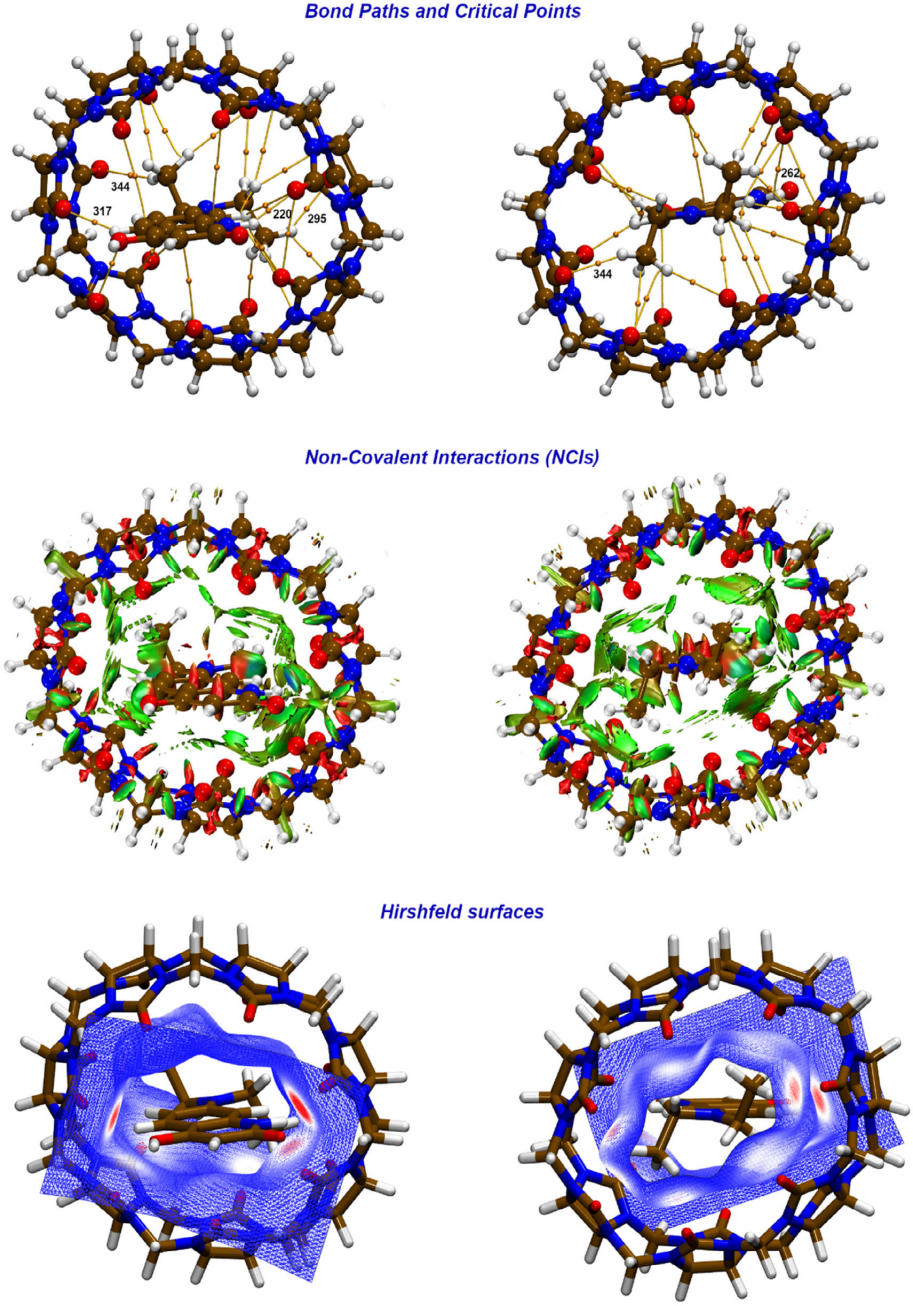
Upper panel: QTAIM noncovalent bond paths and critical points (***r***_**cp**_)—at C‐PCM(H_2_O)/B3LYP(D3)/6‐311++G** level of theory—for the **QD•CB7** supramolecular complex; please note that critical points featuring *ρ*(***r***_**cp**_) ≥ 0.01 *e*·*a*_0_^−3^ are highlighted (see analytical data explicitly collected in Table S2). Middle panel: 3D Noncovalent Interactions (NCIs, RDG isovalue = 0.7 a.u.; lower panel) 3D Hirshfeld surfaces (mapped with *d*_norm_, isovalue = 0.5) associated with **QD** and **CB7** molecular contact points: red zone corresponds to high electron density while blue zone to low electron density.

The recorded time fluorescence profile for **QD** and **QD•CB7** systems is explicitly reported in Figure 7b,c. The photophysical results evidence that the $\phi_{f}$ increases almost 5 times in the supramolecular complex (see Figure S9) regarding the free **QD** probe (0.56 vs. 0.12), which is well correlated with the systematic emission increment displayed in Figure 7a. We observed—according to DFT‐based computations reported in Subsection 3.2—for the free **QD** a fluorescence lifetime dominated by a single component (≈96%) featuring a ***t***_F_ of around 0.59 ns. This can be rationalized by the formation in silico of a TICT excited state (see Figure 6), which should be low‐emissive in the current context. Interestingly, after the inclusion of the probe in **CB7**, we observed an inverse spectroscopical trend indicating a much slower excited state depopulation (***t***_F_ = 4.87 ns) with a contribution of 99.0% in the experimentally derived photophysical properties. These suggest that the macrocycle should plausibly inhibit the formation of a TICT state in the **QD•CB7** system at nanoscale level, thus actually promoting an emissive locally excited state. Looking at recent literature, the fact that ***t***_F_ is longer in the complex (4.87 vs. 0.59 ns) is most likely ascribed to the fact that supramolecular hosting (or even matrices) can rigidify the fluorophore, limiting non‐radiative decay pathways (e.g., vibrational relaxation) and favoring fluorescence, thus extending the lifetime of excited states [34, 82]. TICT processes also contribute to the fluorescence quenching of some quinoline‐based fluorescent probes and N‐alkylated rhodamine dyes, as reported in literature: [83, 84]. Also, taking as reference condition the supramolecular geometry displayed in Figure 8, the presence of a lowest energy Franck‐Condon excited state lying at 360 nm (*f* = 0.32) is seen to be in line with the experimental value recorded at 361 nm (see Figure S10). Furthermore, we also estimated—at C‐PCM(H_2_O)/B3LYP level—for this excitation a net electronic charge of 0.40*e* transferred from fragment 2 → fragment 1 (CT% = 51,69; LE% = 48,31, see Subsection 3.2) during the absorption of light; the contribution of the **CB7** macrocycle (as fragment 3) was found to be negligible.

The supramolecular patterns stabilizing the **QD•CB7** adduct in water solution are characterized by QTAIM descriptors derived from the converged electronic wavefunction at C‐PCM(H_2_O)/B3LYP(D3)/6‐311++G** level of theory. To this end, the QTAIM descriptors are collected in Table S2 and graphically visualized in Figure 8 in terms of Bond Paths (BPs) and Bond Critical Points (BCPs), NCI and Hirshfeld isosurfaces. The investigated supramolecular structure is extracted from a preliminary MD sampling in water at 298 K; we relaxed this adduct at C‐PCM/B3LYP(D3)/6‐311++G** level, with the constraint of maintaining the reciprocal distance between **QD** and **CB7** geometrical centers. The region between the **QD** probe and the macrocycle includes numerous *ρ*(***r***) BCPs of the type (+3,−1) [67, 68, 69, 70, 71]. (A “critical point” (CP) in the electron density is a point in space at which the first derivatives of the density vanish (i.e., ∇*ρ*(***r***) *=* **0,** where the zero vector signifies that each individual derivative in the gradient operator, ∇, is zero and not just their sum). In this table, we also report: *(i)* the trace of the Hessian of the density, known as the Laplacian of the density [$\nabla^{2}$ *ρ*(***r***)]; this parameter allows an analysis of the curvatures of the *ρ*(***r***) at any estimated BCPs along the principal axes. *(ii)* The analytical values of the *G*(***r***) and *V*(**r**) to compare the kinetic and potential energy densities; this to appreciate if interactions are dominated by a local reduction of the potential energy or by a local excess in the kinetic energy [we always have *G*(***r***) > 0 and *V*(***r***) < 0]. *(iii)* The total energy density—see Equation (7)—which reflects a negative value for interactions with significant sharing of electrons, so to reveal the covalent or partially covalent character of the interactions themselves. As it would have been expected, the QTAIM indices computed for the various BCPs clearly unravel the noncovalent nature of any contact occurring between the two molecular species. As a matter of fact, at any BCP, the *ρ*(***r***) is within 0.0208 *e*·*a*_0_^−3^, the $\nabla^{2}$ *ρ*(***r***) and the *H*(***r***) are positive, and the –*G*(***r***)*/V*(***r***) ratio results greater than one (see Table S2).

Our data also indicate that, at least in the mutual conformation considered, the most effective supramolecular forces involve (with *ρ*(***r***) ≥ 0.01 *e·* *a*_0_^−3^): a short H‐bonding contact featuring the polar N—H group of **QD** in interaction with a **CB7** carbonyl groups [i.e., **QD**‐N—H—O—C(sp^2^)‐**CB7**, 2.02 Å]. A quick estimation of the binding energy (*B*_E_) characterizing this H‐bond contact‐based on the electronic properties derived from QTAIM analysis according to the *B*_E_(kcal/mol) = −223.08 × *ρ*(***r***_**cp,220**_) + 0.7423 linear equation [85] reports a *B*_E_ = −3.9 kcal/mol. Another H‐bonding contact of the **QD**‐N—H—O—C(sp^2^)‐**CB7** type lies at 2.37 Å with a *ρ*(***r***_**cp,262**_) of 0.0114 *e*·*a*_0_^−3^ and a *B*_E_ = −1.8 kcal/mol. Carbonyl groups of the macrocycle are also involved in non‐covalent interactions with hydrogen atoms of either the aromatic rings or localized over the —N(CH_2_CH_3_)_2_ moiety [(sp^2^)C—H—O—C(sp^2^); (sp^3^)C—H—O—C(sp^2^)]. H atoms of the aforementioned substituent also present BCPs of the **QD**‐(sp^3^)C—H—N‐**CB7** type contributing to the overall stability of the investigated supramolecular assembly. These are typical examples of weak noncovalent contacts of variable nature with *H*(***r***) estimated values *<* 0.002 hartree·*a*_0_^−3^ (see data in Table S2). [67, 68, 69, 70, 71, 74, 75, 76, 77, 78] In this respect, prominent supramolecular contacts between **QD** and **CB7** are also clearly identified by means of 3D NCI and Hirshfeld surfaces, respectively (see Figure 8). In conclusion, it is therefore evident that the experimentally observed host(**CB7**)–guest(**QD**) self‐assembly may effectively arise from a complex and fine balance of supramolecular interactions in solution modulated by thermal effects and nanosolvation pathways. Given the intrinsic complexity of the investigated systems, a more punctual analysis of the driving forces modulating the detected **CB7**‐based molecular recognition should be plausibly carried out with unbiased MD simulations in the presence of both **QD** and **CB7**.

### 3.4 Thermodynamics of the Supramolecular Binding

**FIGURE 9:**
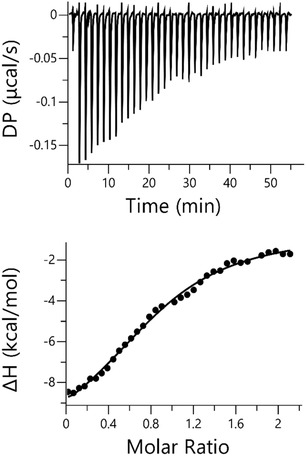
Representative enthalpogram for the binding between **QD** (19 μM) – **CB7** (104 μM) in water:ACN (99:1) mixture at 298 K. Δ*G*(−7.2 ± 0.1 kcal/mol), Δ*H*(−9.9 ± 0.3 kcal/mol), and *T*Δ*S* (−2.7 ± 0.3  kcal/mol) predicted values are estimated averaging over three different measurements (see Table S3 for major details).

To study the thermodynamics of the **QD**•**CB7** complexation, we employed isothermal titration calorimetry, see Figure 9 and Table S3. The results suggest that the binding might be enthalpy driven, which is associated with the liberation of high‐energy water molecules from the inside of the macrocycle cavity [44]. On the other hand, there is a smaller entropy penalization to the binding, which could be attributed to the decrease in the accessible microstates upon the complexation, as the **QD** probe inside the macrocycle tends to sample more ordered nanoscale patterns as a result of the directionally dependent supramolecular interactions driving the **CB7**‐based molecular recognition mechanism. In other words, the spontaneous **QD•CB7** self‐assembly (ΔG = −7.2 ± 0.1 kcal/mol) observed in water:ACN(99:1) mixture at nanoscale level is achieved by favoring enthalpic contributions (Δ*H* = −9.9 ± 0.3 kcal/mol) over entropic penalties (*T*Δ*S* = −2.7 ± 0.3 kcal/mol) through specific noncovalent interactions that compensate for the loss of freedom accompanying the complexation. Previous studies also revealed that the enthalpic gain arising from the host–guest supramolecular interactions, as well as the reduction of entropy upon the complexation, may be strongly modulated by several aspects [86, 87, 88, 89]. However, given the low solubility in water, these results should not be considered conclusive. To further validate the presence of a TICT state, we conducted time‐resolved fluorescent and emission/absorption experiments with different solvents at room temperature conditions. In Table S4 and Figures S11−S12, it is shown that low polarity solvents, such as toluene and diethyl ether, promote the locally excited state (*t*_1_) with a null contribution of the TICT eigenstate (*t*_2_). On the other hand, more polar solvents with greater viscosity have a similar contribution from both states. Glycerol exhibits a similar effect to **CB7**, promoting the second life state, and has a similar emission spectrum to the **QD•CB7** supramolecular complex.

### 3.5 QD as Fluorescence IDA in Confined CB7 Space for TA

**FIGURE 10:**
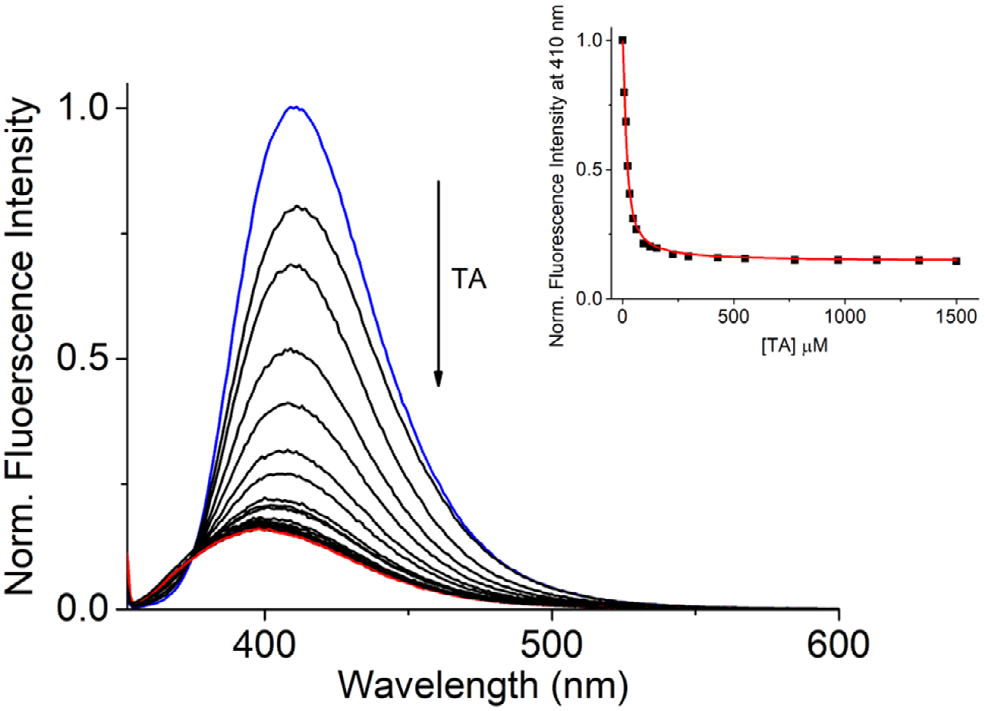
Indicator displacement assay using the complex **QD•CB7** (0.5 and 10 μM respectively) in the presence of an increasing concentration of TA (8–1500 μM). *K*_b,TA•CB7_ = 5.5 × 10^5^ ± 0.2 M^−1^.

To assess the potential use of **QD** as indicator displacement assay, we selected tyramine (**TA**) as a guest antagonist, which is one of the most relevant Biogenic Amines (BAs) due to its physiological effect and high‐dose toxicity [35]; in addition, BAs can accumulate in high concentrations in food due to microbial activity and are typically difficult to prevent in many fermented foods [90]. Thus, contributing to the knowledge of detection methodologies of **TA** is highly appealing to ensure food safety. Always remaining in this context, cucurbituril cyclic compounds had already been applied in the design of electrochemical sensors for the detection of biogenic amines [91]. In this way, we carried out an IDA experimental setup in order to directly evaluate the possibility of determining the binding constant of this amine toward the **CB7** receptor. Figure 10 displays the result of this experiment—in the presence of an increasing concentration of **TA** at room temperature—where the **QD**•**CB7** supramolecular complex with a high fluorescence response (see Figure 10) starts to quench due to the underlying displacement mechanism of **QD** by the **TA** analyte. Afterwards, using Equation (2), we also successfully calculate the binding constant of **TA**•**CB7** supramolecular complex featuring a value of 5.5 × 10^5^ ± 0.2 M^−1^, which is in the same order of binding obtained for modified **CB7** [35, 92]. At last, it should be interesting to remark that the obtained binding properties agree well with data available in literature for many CB[n] host–guest organic analytes in aqueous media (substantially showing binding constants in the range of 10^3^ –10^9^ M^−1^) [93].

## 4 Conclusion

Host–guest complexation of a newly proposed fluorescent probe, 7‐(diethylamino)‐4‐hydroxyquinoline‐2(1H)‐one (**QD**), and its inclusion complex with the Cucurbit [7]uril (**CB7**) macrocycle were studied in solution at room temperature. UV–vis spectroscopic measurements indicate that the observed **QD•CB7** self‐assembly effectively enhances and promotes the photophysical properties of the **QD** system, largely increasing its quantum yield with respect to the derived data in absence of the **CB7** hosting species. Density functional theory (DFT) converged electronic wavefunctions are also self‐consistently analyzed via quantum theory of atoms in molecules (QTAIM) descriptors and within a semiclassical molecular dynamics (MD) picture to derive electronic and structural properties of both aqueous **QD** and **QD•CB7** molecular systems. Interestingly, the picture that has emerged lets us plausibly suppose that, when the guest species is confined within the macrocycle, the encircling environment inhibits the formation of a light‐induced TICT state in the **QD•CB7** system at nanoscale level, thus actually favoring an emissive Franck−Condon locally excited (LE) state. At last, the addition of Tyramine—a primary amine with a high affinity for **CB7**—led to a fluorescence quenching mechanism due to the displacement of the QD dye from the macrocycle. In light of these findings, the synthesized **QD** dye is then proposed as a potential fluorescent indicator‐displacement assay (F‐IDA) for the analytical determination of the binding affinity of biogenic amines in nature.

## Supporting Information

Additional supporting information can be found online in the Supporting Information section. Optimized geometries for the **QD** (Ground state and S1 excited state) and **QD**•**CB7** (Ground state) at C‐PCM(H_2_O)/B3LYP(D3)/6‐311++G** level of theory; vertical excitation transitions of aqueous **QD**; QTAIM descriptors characterizing the supramolecular interaction patterns in the **QD**•**CB7** adduct; isothermal titration calorimetry measurements; ^1^H‐NMR and ^13^C‐NMR spectra of **QD** in DMSO‐d6; UV‐Vis adsorption spectra of the **QD**•**CB7** complex. **Supporting Fig. S1**: ^1^H‐NMR spectra of **QD** in DMSO‐*d*6. **Supporting Fig. S2**: ^13^C‐NMR spectra of **QD** in DMSO‐d6. **Supporting Fig. S3**: DEPT‐135 NMR spectra of **QD** in DMSO‐d6. **Supporting Fig. S4**: HSQC spectra of **QD** in DMSO‐d6. **Supporting Fig. S5**: HMBC spectra of **QD** in DMSO‐d6. **Supporting Fig. S6**: A) MS spectra on positive mode of **QD**. B) HR‐MS spectra on negative mode of **QD**. **Supporting Fig. S7**: Radial pair correlation distribution functions (RDFs, **QD** molecule) as extracted from the classical MD sampling (200 ns, 298K): a) gN‐H–Ow(r); b) O‐H–Ow(r); c) gC=O–Hw1,Hw2(r) In the same graph, the number of water molecules as extracted from RDFs analysis are also reported. **Supporting Fig. S8**: Electron(blue)‐hole(green) distributions of the first electronic excited state of aqueous **QD** at either the ground state (a) or excited state (b) optimized geometry at C‐PCM/B3LYP(D3)/6‐ 311++G** level of computation. **Supporting Fig. S9**: Integrated fluorescence intensity *vs* absorbance for quinine sulfate (black dots) and A) quinolinone derivative (red dots) B) complex **QD**•**CB7** (red dots) used for the determination of quantum yields. **Supporting Fig. S10**: Absorbance for **QD** (black line) and **QD**•**CB7** (red line) supramolecular complex in water/DMSO mixture (99/1) at room temperature, respectively. **Supporting Fig. S11**: UV‐Vis spectra of **QD** in solvents with varying polarities at room temperature. **Supporting Fig. S12**: Emission spectra of **QD** in solvents with varying polarities at room temperature. **Supporting Table S1a**: Singlet electronic excitations (**λ_max_**, in nm) ‐ at C‐PCM(H_2_O)/B3LYP(D3)/6‐311++G** level of theory ‐ of aqueous 7‐(diethylamino)‐4‐hydroxyquinolin‐2(1*H*)‐one (**QD**); the corresponding oscillator strengths (*f*) are reported in a.u.; please note that, the orbitals contributions lower than 10% for each excitation are considered as ‘minor contributions’. **Supporting Table S1b**: Singlet electronic excitations (**λ_max_**, in nm) ‐ at C‐PCM(H_2_O)/B3LYP(D3)/6‐311++G** level of theory ‐ of aqueous 7‐(diethylamino)‐4‐hydroxyquinolin‐2(1*H*)‐one (**QD**); the corresponding oscillator strengths (*f*) are reported in a.u.; please note that, the orbitals contributions lower than 10% for each excitation are considered as ‘minor contributions’. **Supporting Table S2**: Electron density *ρ*(***r***) (*e·a_0_* ^‐3^), Laplacian of electron density ∇^2^ *ρ*(***r***) (*e·a_0_* ^‐5^), electron kinetic energy density *G*(***r***) (hartree·*a_0_* ^‐3^), electron potential energy density *V*(***r***) (hartree·*a_0_* ^‐3^), and electron energy density *H*(**r**) (hartree·*a_0_^‐3^*) for bond critical points on selected bonds of the **QD·CB7** supramolecular assembly calculated at C‐PCM(H_2_O)/B3LYP(D3)/6‐311++G** level of theory. **Supporting Table S3**: Thermodynamic parameters for **QD•CB7** adduct. **Supporting Table S4**: Properties of solvent used and their effect on photophysical properties of **QD**.

## Conflicts of Interest

The authors declare no conflicts of interest.

## Supporting information

Supplementary Material

## Data Availability

The data that support the findings of this study are available in the supplementary material of this article.
