# Supplementary material for: On a Fluorescent Dye for Indicator Displacement From Cucurbit[7]uril‐Based Molecular Recognition: A Joint Experimental‐Computational Study
Source: Chemphyschem. 2026 Mar 31;27(6):e202500620. doi: 10.1002/cphc.202500620 (PMC13036712; doi:10.1002/cphc.202500620)
Supplement: Supplementary file 1 — Supplementary Material [file CPHC-27-e202500620-s001.pdf]

Supporting Information:
On a Fluorescent Dye for Indicator Displacement from Cucurbit[7]uril-Based Molecular Recognition: a joint Experimental-Computational Study

Kevin Droguett, [a] Angélica Fierro, [b] Mario Aranda, [c] Costantino Zazza*,[d] and Margarita E. Aliaga*,[a]

[a] Dr. Kevin Droguett and Prof. Margarita E. Aliaga,
Departamento de Química Física, Escuela de Química, Facultad de Química y de Farmacia,
Pontificia Universidad Católica de Chile, Santiago, Chile.
E-mail: mealiaga@uc.cl

[b] Prof. Angélica Fierro,
Departamento de Química Orgánica, Escuela de Química, Facultad de Química y de Farmacia, Pontificia Universidad Católica de Chile, Chile.
Pontificia Universidad Católica de Chile, Santiago, Chile.

[c] Prof. Mario Aranda,
Departamento de Farmacia, Escuela de Química y Farmacia, Facultad de Química y de Farmacia, Pontificia Universidad Católica de Chile, Chile.
Pontificia Universidad Católica de Chile, Santiago, Chile.

[d] Dr. Costantino Zazza,
Department for Innovation in Biological, Agro-food and Forest systems (DIBAF),
Università degli Studi della Tuscia,
L.go dell'Università, s.n.c., 01100 Viterbo, Italy.
E-mail: costantino.zazza@unitus.it

Content

Table S1a. Singlet electronic excitations (**λ_{max}**, in nm) - at C-PCM(H_2O)/B3LYP(D3)/6-311++G** level of theory - of aqueous 7-(diethylamino)-4-hydroxyquinolin-2(1*H*)-one (**QD**); the corresponding oscillator strengths (*f*) are reported in a.u.; please note that, the orbitals contributions lower than 10% for each excitation are considered as “minor contributions”. 2

Table S1b. Singlet electronic excitations (**λ_{max}**, in nm) - at C-PCM(H_2O)/B3LYP(D3)/6-311++G** level of theory - of aqueous 7-(diethylamino)-4-hydroxyquinolin-2(1*H*)-one (**QD**); the corresponding oscillator strengths (*f*) are reported in a.u.; please note that, the orbitals contributions lower than 10% for each excitation are considered as “minor contributions”. 3

Table S2. Electron density $\rho(\mathbf{r})$ ($e \cdot a_0^{-3}$), Laplacian of electron density $\nabla^2\rho(\mathbf{r})$ ($e \cdot a_0^{-5}$), electron kinetic energy density $G(\mathbf{r})$ (hartree$\cdot a_0^{-3}$), electron potential energy density $V(\mathbf{r})$ (hartree$\cdot a_0^{-3}$), and electron energy density $H(\mathbf{r})$ (hartree$\cdot a_0^{-3}$) for bond critical points on selected bonds of the **QD·CB7** supramolecular assembly calculated at C-PCM(H_2O)/B3LYP(D3)/6-311++G** level of theory. 6

Table S3. Thermodynamic parameters for **QD•CB7** adduct. 9

Figure S1. ^{1}H-NMR spectra of **QD** in DMSO-*d*6. 9

Figure S2. ^{13}C-NMR spectra of **QD** in DMSO-d6. 10

Figure S3. DEPT-135 NMR spectra of **QD** in DMSO-d6. 10

Figure S4. HSQC spectra of **QD** in DMSO-d6. 11

Figure S5. HMBC spectra of **QD** in DMSO-d6. 11

Figure S6. A) MS spectra on positive mode of **QD**. B) HR-MS spectra on negative mode of **QD**. 12

Figure S7. Radial pair correlation distribution functions (RDFs) as extracted from the classical MD sampling (200ns, 298K): a) gN-H–O_w(r); b) O-H–O_w(r); c) gC=O–H_{w1},H_{w2}(r) In the same graph, the number of water molecules as extracted from RDFs analysis are also reported. 13

Figure S8. Electron(blue)-hole(green) distributions of the first electronic excited state of aqueous QD at either the ground state (a) or excited state (b) optimized geometry at C-PCM/B3LYP(D3)/6-311++G** level of computation. 14

Figure S9. Integrated fluorescence intensity *vs* absorbance for quinine sulfate (black dots) and A) quinolinone derivative (red dots) B) complex **QD•CB7** (red dots) used for the determination of quantum yields. 14

Figure S10. Absorbance for **QD** (black line) and **QD•CB7** (red line) supramolecular complex in water/DMSO mixture (99/1) at room temperature, respectively. 15

Figure S11. UV-Vis spectra of **QD** in solvents with varying polarities at room temperature. 15

Figure S12. Emission spectra of **QD** in solvents with varying polarities at room temperature 16

Table S4. Properties of solvent used and their effect on photophysical properties of **QD**. 16

Supporting Information:

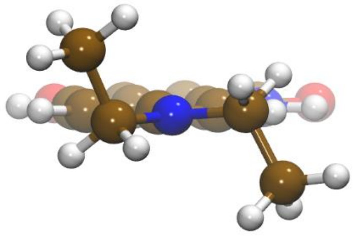

Table S1a. Singlet electronic excitations (**λ$_{max}$**, in nm) - at C-PCM(H_2O)/B3LYP(D3)/6-311++G** level of theory - of aqueous 7-(diethylamino)-4-hydroxyquinolin-2(1*H*)-one (**QD**); the corresponding oscillator strengths (*f*) are reported in a.u.; please note that, the orbitals contributions lower than 10% for each excitation are considered as "minor contributions".

λ$_{max}$ (nm)	*f* (a.u.)	Major contributions
335.02	0.4745	HOMO->LUMO (95%)
289.20	0.0279	H-1->LUMO (44%), HOMO->L+1 (51%)
270.44	0.1802	H-1->LUMO (52%), HOMO->L+1 (41%)
268.85	0.0199	HOMO->L+2 (95%)
252.83	0.0088	HOMO->L+3 (98%)
249.93	0.0182	HOMO->L+4 (96%)
247.74	0	H-3->LUMO (93%)
242.46	0.0008	H-2->LUMO (11%), HOMO->L+5 (83%)
236.79	0.1156	H-2->LUMO (53%), HOMO->L+6 (25%)
234.06	0.0176	HOMO->L+6 (25%), HOMO->L+7 (70%)
230.75	0.2194	H-2->LUMO (13%), HOMO->L+6 (41%), HOMO->L+7 (20%), HOMO->L+8 (10%)
225.70	0.0141	H-1->L+2 (65%), HOMO->L+8 (19%)
223.66	0.1205	H-1->L+1 (16%), H-1->L+2 (24%), HOMO->L+8 (47%)
221.39	0.6462	H-1->L+1 (65%), HOMO->L+8 (21%)
218.28	0.0038	HOMO->L+9 (78%)
212.59	0.0476	H-1->L+4 (23%), HOMO->L+10 (57%)
211.22	0.0282	H-1->L+3 (43%), H-1->L+4 (33%), HOMO->L+10 (10%)
210.08	0.0234	H-2->L+1 (73%), H-1->L+3 (15%)
209.75	0.0619	H-2->L+1 (13%), H-1->L+3 (37%), H-1->L+4 (24%), HOMO->L+10 (11%)
209.13	0.0161	HOMO->L+10 (10%), HOMO->L+11 (71%)
207.84	0.0899	H-1->L+5 (53%), HOMO->L+12 (21%)
207.41	0.0158	H-1->L+5 (24%), HOMO->L+12 (65%)
204.33	0.0002	H-3->L+1 (86%)
204.07	0.0112	HOMO->L+13 (97%)
203.20	0.0008	H-2->L+2 (73%), H-2->L+3 (18%)
199.98	0.0035	H-4->LUMO (68%), H-1->L+6 (17%)
197.42	0.0065	H-1->L+7 (86%)
196.07	0.0388	H-4->LUMO (16%), H-1->L+6 (69%)
195.58	0.0139	HOMO->L+14 (92%)
192.60	0.0099	H-1->L+8 (18%), HOMO->L+15 (53%), HOMO->L+16 (12%)

33

H	-0.007038	0.036000	0.019172
O	0.017371	0.034644	0.984608
C	1.298838	0.011568	1.425544
C	2.375705	-0.007013	0.579121
H	2.251850	-0.003717	-0.496218
C	3.721281	-0.032423	1.086377
O	4.746554	-0.050638	0.383952
N	3.837003	-0.035567	2.470020
H	4.783652	-0.050827	2.827591
C	2.781416	-0.016145	3.364030
C	1.464467	0.006102	2.848893
C	0.401486	0.021163	3.773171
H	-0.614510	0.046111	3.399775
C	0.624093	0.015219	5.130110
H	-0.227739	0.048383	5.792837
C	1.951945	-0.009939	5.660530
C	3.018864	-0.027851	4.738688
H	4.044468	-0.069209	5.076316
N	2.173280	-0.012411	7.012435
C	1.080083	-0.118971	7.983045
H	1.482937	-0.613954	8.869738
H	0.308524	-0.786705	7.595085
C	0.482099	1.236503	8.370727
H	1.248133	1.886154	8.802237
H	-0.310760	1.103682	9.111991
H	0.058466	1.742574	7.500181
C	3.522492	0.084292	7.574318
H	3.433820	0.564905	8.551308
H	4.124320	0.760338	6.962782
C	4.213843	-1.274449	7.724569
H	4.322360	-1.770980	6.757684
H	3.634374	-1.929854	8.380050
H	5.208678	-1.147748	8.160258

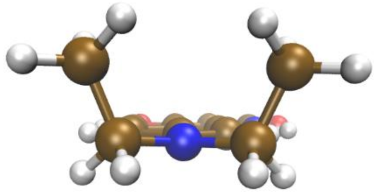

Table S1b. Singlet electronic excitations (**λ_{max}**, in nm) - at C-PCM(H_2O)/B3LYP(D3)/6-311++G** level of theory - of aqueous 7-(diethylamino)-4-hydroxyquinolin-2(1*H*)-one (**QD**); the corresponding oscillator strengths (*f*) are reported in a.u.; please note that, the orbitals contributions lower than 10% for each excitation are considered as "minor contributions".

λ_{max} (nm)	*f* (a.u.)	Major contributions
334	0.48	HOMO->LUMO (95%)
289	0.02	H-1->LUMO (49%), HOMO->L+1 (46%)
270	0.17	H-1->LUMO (43%), HOMO->L+1 (45%)
266	0.02	HOMO->L+2 (89%)
251	0.02	HOMO->L+3 (95%)
248	0.00	H-3->LUMO (93%)
245	0.01	HOMO->L+4 (56%), HOMO->L+5 (34%)
244	0.01	HOMO->L+4 (41%), HOMO->L+5 (52%)
237	0.13	H-2->LUMO (62%), HOMO->L+7 (12%)

233	0.04	HOMO->L+6 (70%), HOMO->L+7 (25%)
229	0.14	H-2->LUMO (11%), HOMO->L+6 (16%), HOMO->L+7 (57%)
226	0.01	H-1->L+2 (46%), HOMO->L+8 (34%)
224	0.07	H-1->L+2 (27%), HOMO->L+8 (62%)
222	0.71	H-1->L+1 (67%), H-1->L+2 (16%)
218	0.03	HOMO->L+9 (86%)
212	0.09	H-1->L+3 (59%), H-1->L+5 (20%)
210	0.04	H-2->L+1 (39%), H-1->L+3 (15%), H-1->L+4 (23%), HOMO->L+10 (13%)
210	0.01	H-2->L+1 (49%), H-1->L+4 (32%)
209	0.03	H-1->L+4 (20%), HOMO->L+10 (62%)
207	0.05	H-1->L+3 (12%), H-1->L+5 (42%), HOMO->L+11 (24%)
207	0.01	HOMO->L+12 (87%)
206	0.02	H-1->L+5 (20%), HOMO->L+11 (69%)
205	0.00	H-3->L+1 (85%)
203	0.01	HOMO->L+13 (92%)
203	0.00	H-2->L+2 (74%), H-2->L+3 (13%)
200	0.00	H-4->LUMO (68%), H-1->L+6 (10%)
197	0.00	H-1->L+6 (47%), H-1->L+7 (43%)
196	0.05	H-4->LUMO (17%), H-1->L+6 (33%), H-1->L+7 (39%)
194	0.00	HOMO->L+14 (90%)
193	0.00	H-1->L+8 (91%)

Optimized geometry (XYZ format, Ground State) of the **QD** molecular system calculated at C-PCM(H_2O)/B3LYP(D3)/6-311++G** level of theory.

33

C	-5.271271	-0.191499	0.494556
C	-4.117956	-0.179321	1.233174
C	-3.097575	0.802807	1.011990
C	-3.313803	1.769598	0.003407
C	-5.507342	0.785438	-0.534740
H	-1.710633	0.150168	2.520851
C	-1.893774	0.876432	1.739023
C	-2.366829	2.759123	-0.263260
H	-4.660433	2.405962	-1.438933
C	-1.156574	2.822065	0.457243
C	-0.953670	1.847238	1.483654
H	-2.592648	3.477957	-1.037284
H	-0.053862	1.859664	2.079631
N	-4.494775	1.717969	-0.715382
O	-6.526892	0.839599	-1.243360
N	-0.201286	3.768451	0.177606
C	1.014618	3.887535	0.990212
H	1.746954	4.430027	0.390375
C	-0.439339	4.803722	-0.832941
H	0.536134	5.184922	-1.139108
H	-0.873162	4.340277	-1.723155
H	1.438216	2.893346	1.152630
C	-1.319235	5.966293	-0.356291
H	-0.836726	6.513524	0.455838
H	-1.494707	6.663060	-1.180648
H	-2.287855	5.610870	0.001810
C	0.814494	4.605801	2.330132
H	1.752865	4.615482	2.891686
H	0.498190	5.639365	2.176471
H	0.057900	4.105580	2.938682
H	-6.042928	-0.932611	0.658840
O	-3.860272	-1.080704	2.211847
H	-4.594174	-1.702028	2.301091

Optimized geometry (XYZ format, Locally Excited S_1) of the **QD** molecular system calculated at C-PCM(H_2O)/B3LYP(D3)/6-311++G** level of theory.

33

H	0.000000	0.000000	0.000000
O	0.000000	0.000000	0.964283
C	1.291654	0.000000	1.431665
C	2.403690	0.022802	0.573128
H	2.274945	0.042571	-0.502460
C	3.729212	0.018979	1.074091
O	4.788450	0.029676	0.396589
N	3.838840	0.000554	2.478856
H	4.783099	-0.011610	2.839677
C	2.779383	-0.031069	3.365714
C	1.444603	-0.020551	2.843769
C	0.363524	-0.027678	3.771429
H	-0.648372	-0.010525	3.391875
C	0.591058	-0.068820	5.148614
H	-0.259466	-0.085717	5.813571
C	1.904859	-0.095462	5.655699
C	3.000615	-0.064150	4.732378
H	4.020492	-0.056324	5.086977
N	2.163485	-0.095880	7.018513
C	1.112692	0.226527	7.982931
H	1.441077	-0.137418	8.955653
H	0.201774	-0.323153	7.734333
C	0.827693	1.734592	8.046565
H	1.720508	2.282233	8.355268
H	0.033833	1.921819	8.773472
H	0.505244	2.106874	7.072541
C	3.489150	-0.401768	7.559603
H	3.512876	-0.028448	8.582614
H	4.255591	0.147142	7.010107
C	3.791885	-1.907559	7.536064
H	3.785192	-2.285157	6.511636
H	3.050767	-2.459361	8.118254
H	4.779764	-2.084761	7.968457

Optimized geometry (XYZ format, Non-Local Excited S_1) of the **QD** molecular system calculated at C-PCM(H_2O)/B3LYP(D3)/6-311++G** level of theory.

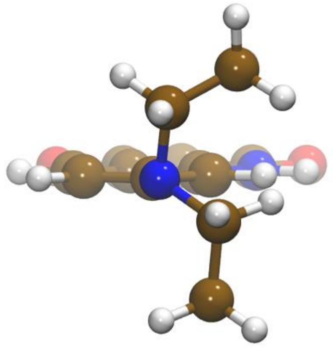

33

H	0.000000	0.000000	0.000000
O	0.000000	0.000000	0.965937
C	1.267199	0.000000	1.438868
C	2.368008	-0.001008	0.631233
H	2.278389	-0.002335	-0.447736
C	3.703206	0.001357	1.176579
O	4.742700	0.002697	0.500067
N	3.777176	0.000149	2.561519
H	4.711993	-0.001724	2.949887
C	2.693742	0.002235	3.418895
C	1.391337	0.005854	2.875022
C	0.291477	0.016931	3.753914
H	-0.708956	0.016520	3.341057
C	0.483285	0.012627	5.120139
H	-0.362436	0.006244	5.797009

C	1.785006	0.001905	5.666686
C	2.880428	0.020552	4.808459
H	3.888829	0.033643	5.202463
N	1.906428	0.054992	7.087816
C	2.046655	1.436326	7.605353
H	1.909426	1.385225	8.689512
H	1.210737	2.019165	7.209009
C	3.365876	2.147129	7.279321
H	4.224398	1.626861	7.710666
H	3.346478	3.159662	7.691990
H	3.518354	2.225156	6.199964
C	3.018093	-0.716482	7.669821
H	2.982531	-0.539093	8.748294
H	4.005203	-0.364340	7.335334
C	2.884927	-2.212671	7.397410
H	2.938575	-2.431625	6.328158
H	1.930831	-2.589005	7.775555
H	3.693763	-2.756788	7.891944

Table S2. Electron density $\rho(\boldsymbol{r})$ ($e\cdot a_0^{-3}$), Laplacian of electron density $\nabla^2\rho(\boldsymbol{r})$ ($e\cdot a_0^{-5}$), electron kinetic energy density $G(\boldsymbol{r})$ (hartree$\cdot a_0^{-3}$), electron potential energy density $V(\boldsymbol{r})$ (hartree$\cdot a_0^{-3}$), and electron energy density $H(\mathbf{r})$ (hartree$\cdot a_0^{-3}$) for bond critical points on selected bonds of the **QD·CB7** supramolecular assembly calculated at C-PCM(H_2O)/B3LYP(D3)/6-311++G** level of theory.

BCP [a]	Moieties(BCP)	Distance (Å)	$\rho(\boldsymbol{r})$	$\nabla^2\rho(\boldsymbol{r})$	$G(\boldsymbol{r})$	$V(\boldsymbol{r})$	$-G(\boldsymbol{r})/V(\boldsymbol{r})$	$H(\mathbf{r})$
N-H - O=C(sp^2)	QD-CB7(220)	2.02	0.0208	0.0700	0.0164	-0.0152	1.0748	0.0011
N-H - O=C(sp^2)	QD-CB7(262)	2.37	0.0114	0.0429	0.0098	-0.0089	1.1000	0.0009
(sp^2)C-H - O=C(sp^2)	QD-CB7(317)	2.29	0.0133	0.0416	0.0101	-0.0098	1.0332	0.0003
(sp^2)C-H - O=C(sp^2)	QD-CB7(352)	2.81	0.0054	0.0224	0.0044	-0.0032	1.3786	0.0012
(sp^2)C-H - O=C(sp^2)	QD-CB7(286)	2.93	0.0043	0.0180	0.0035	-0.0025	1.4031	0.0010
(sp^2)C-H - O=C(sp^2)	QD-CB7(233)	2.97	0.0039	0.0159	0.0031	-0.0022	1.4176	0.0009
(sp^2)HC - O=C(sp^2)	QD-CB7(254)	3.62	0.0028	0.0095	0.0019	-0.0014	1.3521	0.0005
(sp^2)HC - O=C(sp^2)	QD-CB7(335)	3.67	0.0018	0.0075	0.0014	-0.0010	1.4287	0.0004
(sp^2)HC - O=C(sp^2)	QD-CB7(212)	3.89	0.0020	0.0071	0.0014	-0.0010	1.4278	0.0004
(sp^3)C-H - O=C(sp^2)	QD-CB7(295)	2.47	0.0105	0.0331	0.0078	-0.0072	1.0713	0.0005
(sp^3)C-H - O=C(sp^2)	QD-CB7(377)	2.57	0.0085	0.0266	0.0061	-0.0055	1.1004	0.0006
(sp^3)C-H - O=C(sp^2)	QD-CB7(336)	2.61	0.0097	0.0383	0.0079	-0.0064	1.2367	0.0015
(sp^3)C-H - O=C(sp^2)	QD-CB7(259)	2.78	0.0052	0.0178	0.0038	-0.0032	1.1991	0.0006
(sp^3)C-H - O=C(sp^2)	QD-CB7(285)	2.85	0.0059	0.0239	0.0047	-0.0034	1.3840	0.0013
(sp^3)C-H - C(sp^2)	QD-CB7(379)	2.73	0.0059	0.0240	0.0046	-0.0031	1.4539	0.0014
(sp^3)C-H - C(sp^2)	QD-CB7(273)	3.26	0.0051	0.0217	0.0039	-0.0025	1.6045	0.0015
(sp^3)C-H - O=C(sp^2)	QD-CB7(344)	2.41	0.0118	0.0345	0.0084	-0.0082	1.0251	0.0002
(sp^3)C-H - O=C(sp^2)	QD-CB7(258)	2.74	0.0058	0.0195	0.0042	-0.0036	1.1723	0.0006
(sp^3)C-H - O=C(sp^2)	QD-CB7(375)	2.85	0.0053	0.0213	0.0042	-0.0031	1.3502	0.0011
(sp^3)C-H - N	QD-CB7(338)	2.83	0.0060	0.0179	0.0040	-0.0035	1.1440	0.0005
(sp^3)C-H - N	QD-CB7(237)	3.04	0.0041	0.0141	0.0029	-0.0021	1.3461	0.0007
(sp^3)C-H - N	QD-CB7(227)	3.16	0.0032	0.0118	0.0023	-0.0017	1.3833	0.0006
(sp^2)C-H - H-C(sp^3)	QD-QD(339)	1.99	0.0152	0.0595	0.0126	-0.0103	1.2252	0.0023
(sp^2)C-H - H-C(sp^3)	QD-QD(265)	2.03	0.0142	0.0564	0.0117	-0.0094	1.2516	0.0024
(sp^2)C=O - O=C(sp^2)	CB7-CB7(273)	3.24	0.0055	0.0221	0.0047	-0.0039	1.2132	0.0008

Optimized geometry (XYZ format) of the **QD·CB7** supramolecular assembly calculated at C-PCM(H_2O)/B3LYP(D3)/6-311++G** level of theory.

		159	
C	1.284084	0.492607	1.396687
C	0.083172	0.040684	0.760842
C	-1.005801	-0.319897	1.588953
C	-0.901634	-0.254291	2.985704
C	0.296512	0.197110	3.600880
C	1.374117	0.569817	2.770397
N	-1.966004	-0.611390	3.785188
C	-1.979790	-0.560846	5.173788
C	-0.750198	-0.112383	5.791729
C	0.337318	0.252208	5.035678
N	0.003098	-0.036462	-0.606194
O	1.505803	0.685805	5.573425
O	-3.004162	-0.892431	5.794358
C	-1.175033	-0.562963	-1.289461
C	1.030051	0.526977	-1.481413
C	2.063231	-0.503156	-1.927389
C	-2.192636	0.530924	-1.600265
H	2.154913	0.764720	0.803224
H	-1.957408	-0.632045	1.166169
H	1.574476	-1.376180	-2.386924
H	-2.609344	0.971241	-0.682257
H	-3.007198	0.134054	-2.213691
H	-1.723958	1.340140	-2.180754
H	2.304244	0.915466	3.213810
H	-0.728783	-0.070228	6.881977
H	-0.836297	-1.041314	-2.221589
H	-1.624148	-1.372593	-0.695538
H	0.525282	0.956354	-2.360365
H	1.509746	1.380164	-0.982090
H	2.664001	-0.859979	-1.074971
H	2.746974	-0.072843	-2.670412
H	-2.837263	-0.909819	3.341022
H	1.430392	0.688417	6.541102
C	-0.498175	-4.458489	1.467136
N	-1.583259	-4.712724	0.645484
C	-1.197366	-5.250999	-0.637870
C	0.350169	-5.382828	-0.528820
N	0.648336	-4.776450	0.755997
N	-1.382211	-4.344131	-1.759679
C	-0.181665	-4.044682	-2.389264
N	0.836922	-4.647720	-1.674430
O	-0.545453	-4.055755	2.620376
O	-0.050552	-3.382154	-3.410249
C	-2.945295	-4.694525	1.122085
C	-2.641134	-4.247001	-2.470443
C	-4.089013	-2.251187	-2.530274
N	-3.604091	-3.359999	-1.859425
C	-4.427089	-3.722682	-0.721884
C	-5.489195	-2.578212	-0.667113
N	-5.151712	-1.744502	-1.803756
N	-3.761326	-3.636689	0.564118
C	-4.278784	-2.629350	1.355164
N	-5.264163	-1.988558	0.637584
O	-3.662926	-1.811023	-3.588665
O	-3.940332	-2.380679	2.507545
C	-5.991189	-0.673490	-2.310778
C	3.498672	-3.040054	1.644815
N	4.624317	-2.576057	0.983323
C	4.985764	-3.410708	-0.148666
C	3.769649	-4.380445	-0.270262
N	2.971001	-4.078549	0.897563
N	5.024549	-2.751755	-1.435201
C	3.983820	-3.138240	-2.261590
N	3.175291	-4.004940	-1.541998
O	3.064988	-2.630869	2.711776
O	3.816670	-2.801059	-3.423452
C	-5.055537	1.611238	-2.477983
N	-5.698205	0.626644	-1.751173
C	-6.152544	1.097225	-0.454285
C	-5.518490	2.524360	-0.355149
N	-4.918408	2.717035	-1.657768
N	-5.607140	0.411858	0.696984

C	-4.707466	1.182935	1.404218
N	-4.584993	2.398692	0.755625
O	-4.694345	1.530098	-3.642725
O	-4.142416	0.856156	2.438951
C	-4.320071	3.948630	-2.121856
C	-1.842231	4.054340	-2.352914
N	-2.994080	4.223059	-1.606577
C	-2.736015	4.861787	-0.338692
C	-1.240125	5.276387	-0.432712
N	-0.777602	4.562281	-1.616584
N	-2.751518	3.977328	0.816186
C	-1.572854	4.051425	1.544524
N	-0.681362	4.818660	0.816259
C	-6.025612	-0.885975	1.175527
C	-3.980999	3.528630	1.427156
C	2.172127	-4.805068	-2.207251
C	1.927962	-4.933726	1.413615
C	2.662548	3.979373	1.585329
N	1.740321	4.642750	0.791211
C	2.282491	5.007952	-0.501108
C	3.714821	4.393248	-0.481854
N	3.792840	3.755317	0.815925
N	1.646197	4.366347	-1.637371
C	2.518816	3.534709	-2.323412
N	3.709888	3.498338	-1.620936
O	2.509993	3.674661	2.758646
O	2.282198	2.954708	-3.374379
C	5.074884	0.777433	1.688009
N	5.363581	1.898201	0.933784
C	6.059145	1.574525	-0.290617
C	6.334521	0.043161	-0.158838
N	5.514147	-0.330459	0.985172
N	5.276090	1.653351	-1.517021
C	5.347419	0.478057	-2.246243
N	5.926356	-0.483911	-1.441020
O	4.557922	0.768210	2.796554
O	4.990796	0.328487	-3.406096
C	5.534104	-1.639210	1.600573
C	6.076094	-1.857837	-1.872018
C	5.021662	3.231973	1.375475
C	4.907963	2.896058	-2.160304
C	0.573533	5.279166	1.360307
C	0.466140	4.905653	-2.277460
O	-1.772206	3.563054	-3.470857
O	-1.367095	3.556359	2.642985
H	-1.711556	-6.207204	-0.827395
H	0.709676	-6.424150	-0.557813
H	-4.863178	-4.724639	-0.861886
H	-6.529068	-2.931493	-0.749625
H	5.942674	-3.921714	0.045257
H	4.052607	-5.446116	-0.276123
H	-7.253108	1.099303	-0.411085
H	-6.252106	3.319403	-0.146938
H	-3.423866	5.709491	-0.186843
H	-1.085998	6.361461	-0.549864
H	2.273735	6.103318	-0.626457
H	4.518453	5.139734	-0.589170
H	6.973328	2.182672	-0.382031
H	7.391150	-0.201395	0.036166
H	-3.416895	-5.664737	0.893053
H	-2.909028	-4.541557	2.207000
H	-2.425575	-3.847276	-3.468219
H	-3.081559	-5.255328	-2.558440
H	-5.815979	-0.598424	-3.390722
H	-7.044060	-0.932177	-2.121018
H	-4.238730	3.869332	-3.212213
H	-4.975085	4.792182	-1.853916
H	-7.089472	-1.027592	0.935436
H	-5.887535	-0.903574	2.263039
H	-4.706187	4.360325	1.456215
H	-3.737512	3.213240	2.448753
H	2.447263	-5.872081	-2.145096
H	2.154678	-4.485779	-3.255936
H	1.779820	-4.673140	2.468281
H	2.255917	-5.984239	1.331315
H	5.218066	-1.510034	2.642761

H	6.557401	-2.048962	1.569174
H	7.042898	-2.239697	-1.512614
H	6.061069	-1.854652	-2.968156
H	4.890769	3.195119	2.463155
H	5.852791	3.907839	1.120309
H	4.717605	2.663560	-3.214838
H	5.736831	3.619081	-2.080900
H	0.573656	5.050346	2.432418
H	0.641474	6.370163	1.210479
H	0.419475	4.488773	-3.290159
H	0.561353	6.005470	-2.330907

Table S3. Thermodynamic parameters for **QD•CB7** adduct.

	ΔH (kcal/mol)	-TΔS (kcal/mol)	ΔG (kcal/mol)	KITC
1	-10.5	3.29	-7.21	1.93E+05
2	-9.59	2.45	-7.14	1.71E+05
3	-9.6	2.28	-7.32	2.31E+05
Media	-9.90	2.67	-7.22	1.98E+05

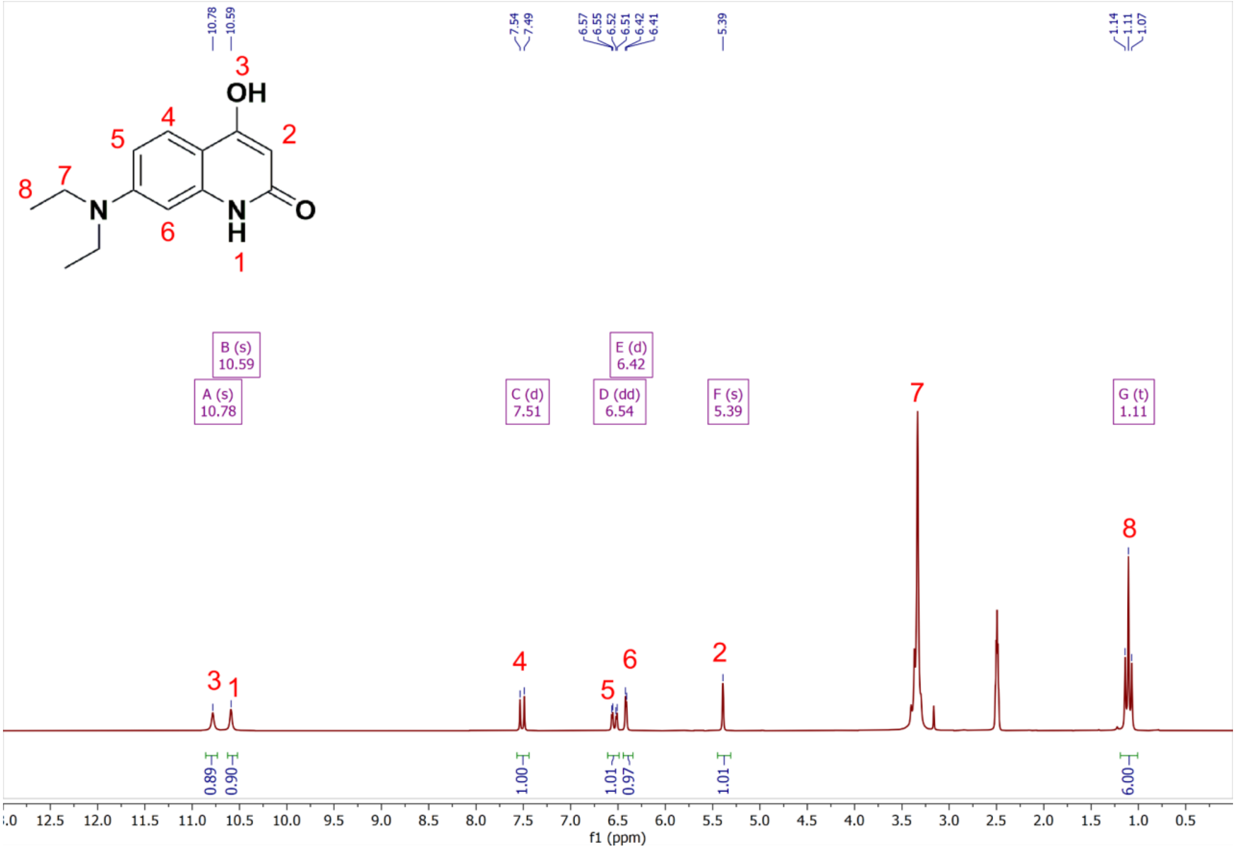

Figure S1. ^{1}H-NMR spectra of **QD** in DMSO-*d*6.

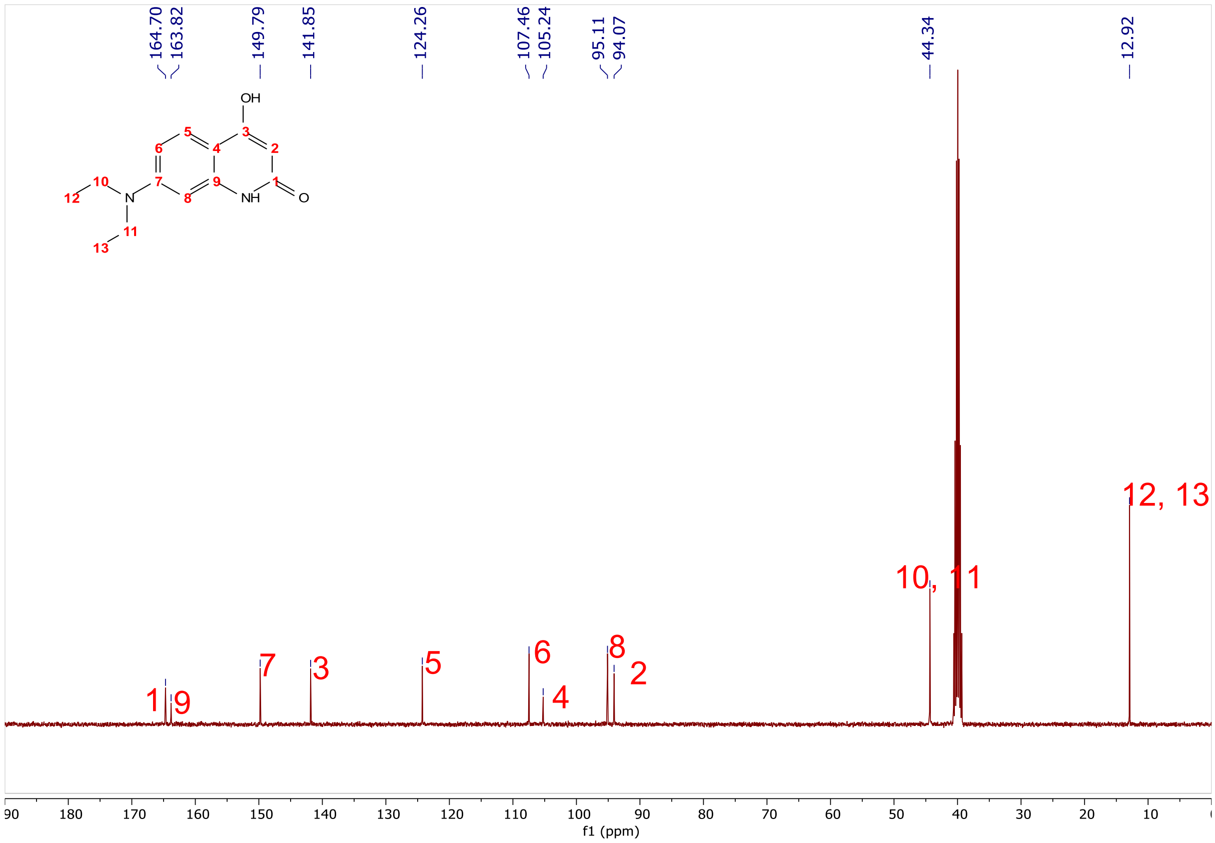

Figure S2. ^{13}C-NMR spectra of **QD** in DMSO-d6.

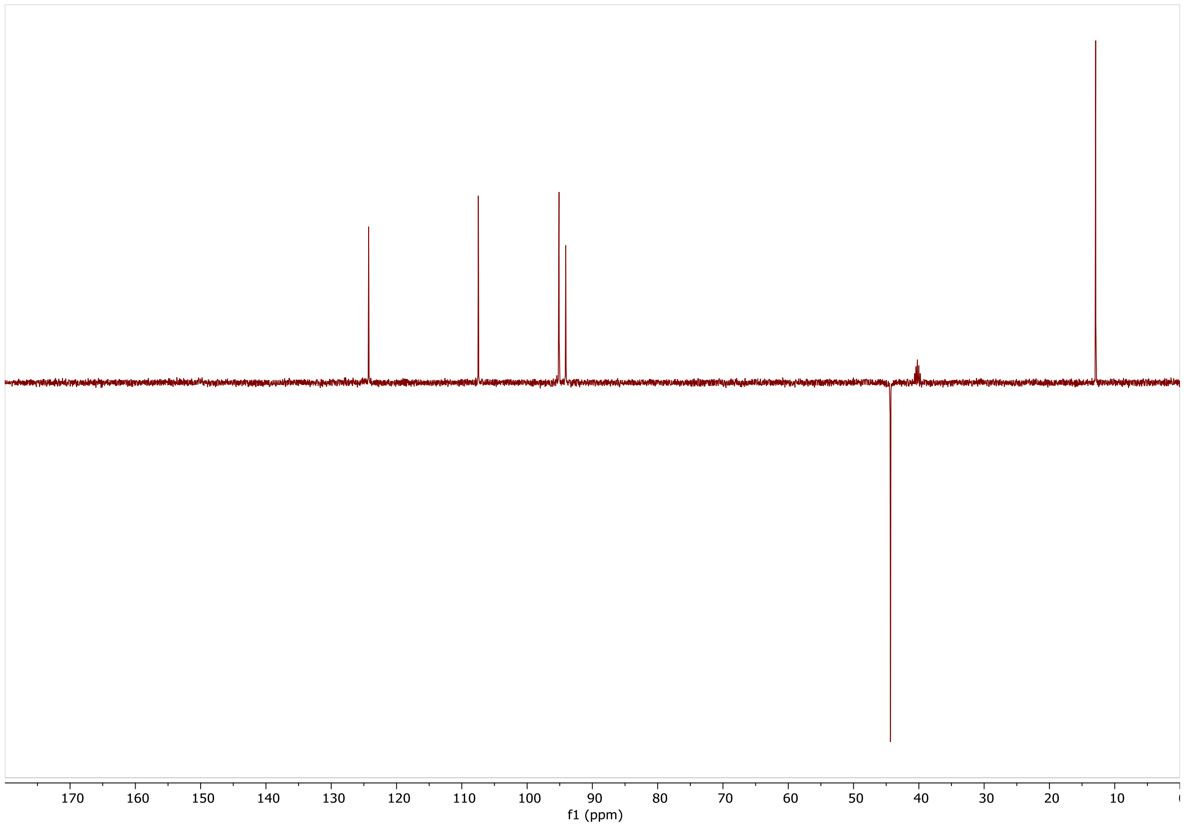

Figure S3. DEPT-135 NMR spectra of **QD** in DMSO-d6.

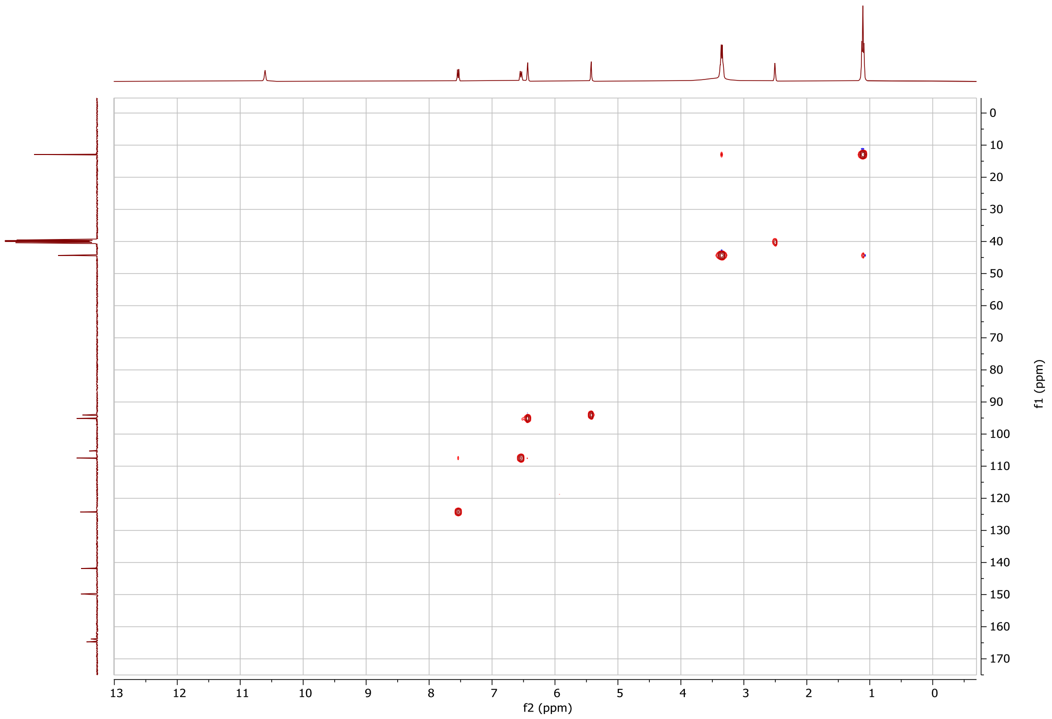

Figure S4. HSQC spectra of **QD** in DMSO-d6.

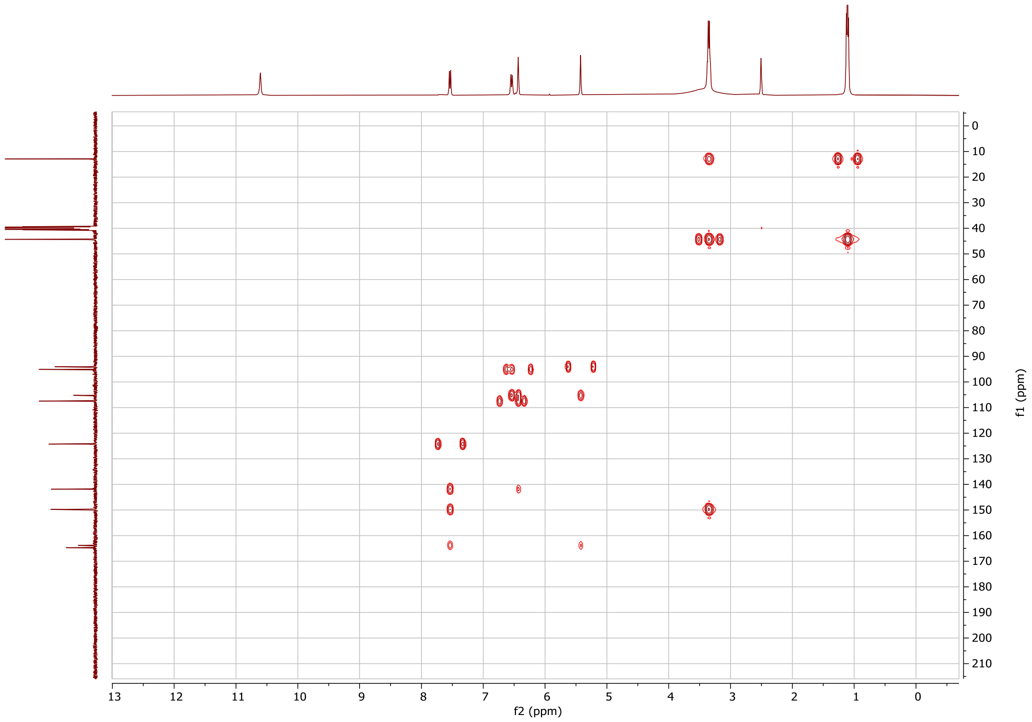

Figure S5. HMBC spectra of **QD** in DMSO-d6.

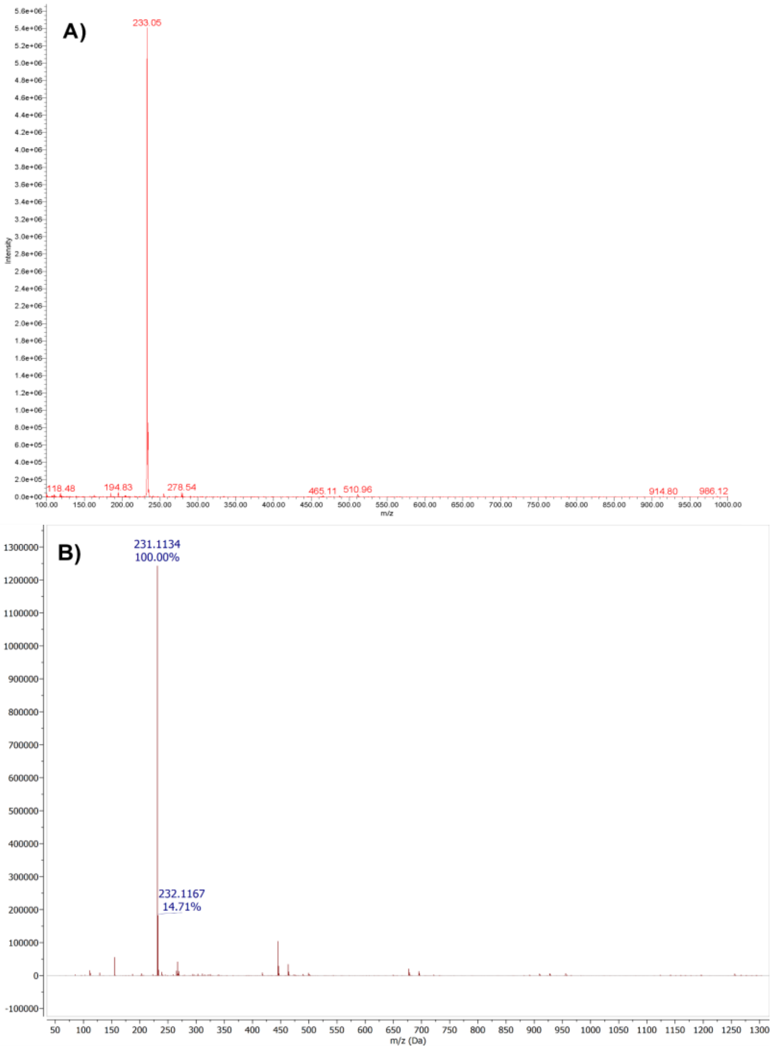

Figure S6. A) MS spectra on positive mode of **QD**. B) HR-MS spectra on negative mode of **QD**.

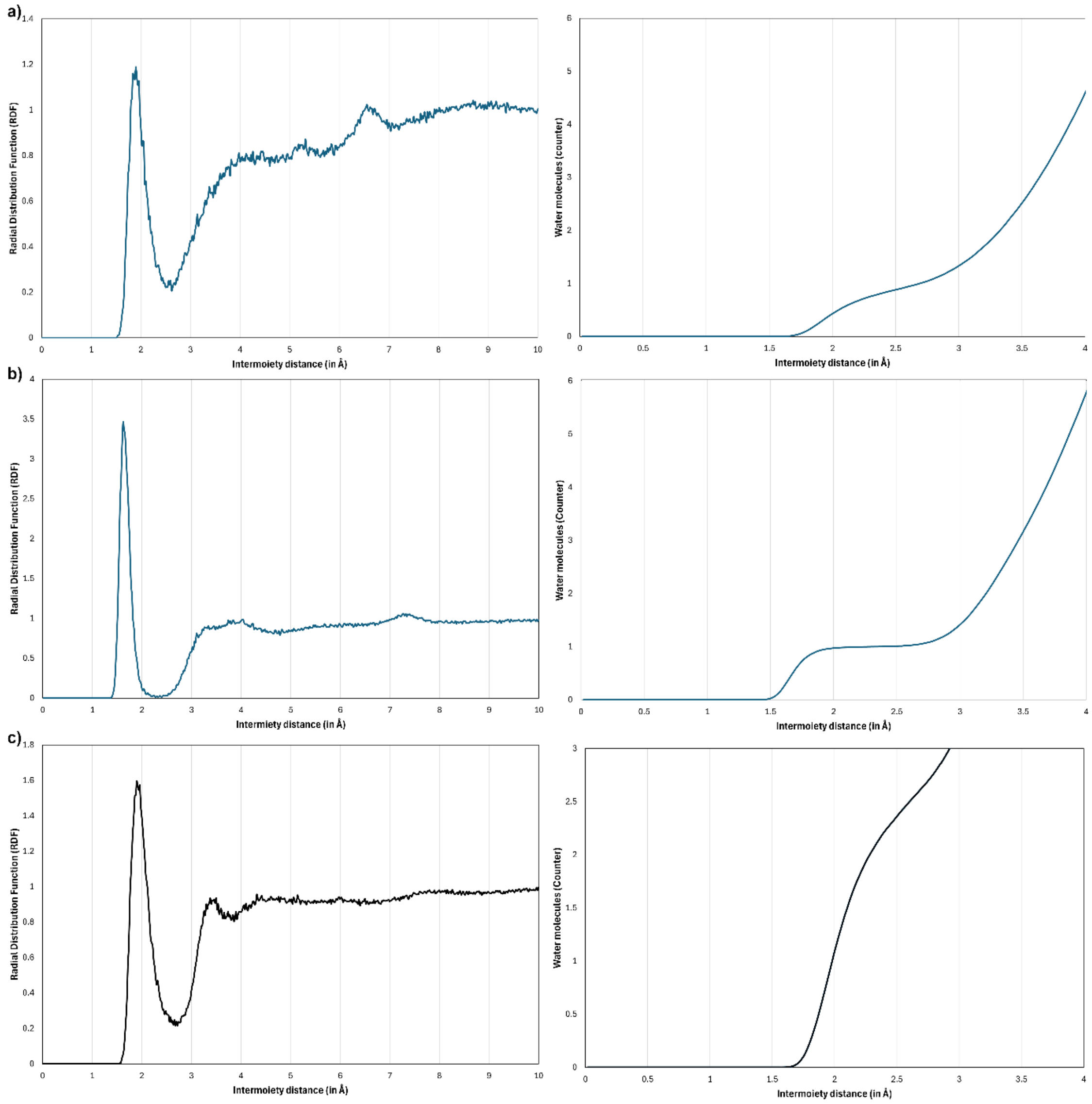

Figure S7. Radial pair correlation distribution functions (RDFs, **QD** molecule) as extracted from the classical MD sampling (200ns, 298K): a) gN-H–O_w(r); b) O-H–O_w(r); c) gC=O–H_{w1},H_{w2}(r) In the same graph, the number of water molecules as extracted from RDFs analysis are also reported.

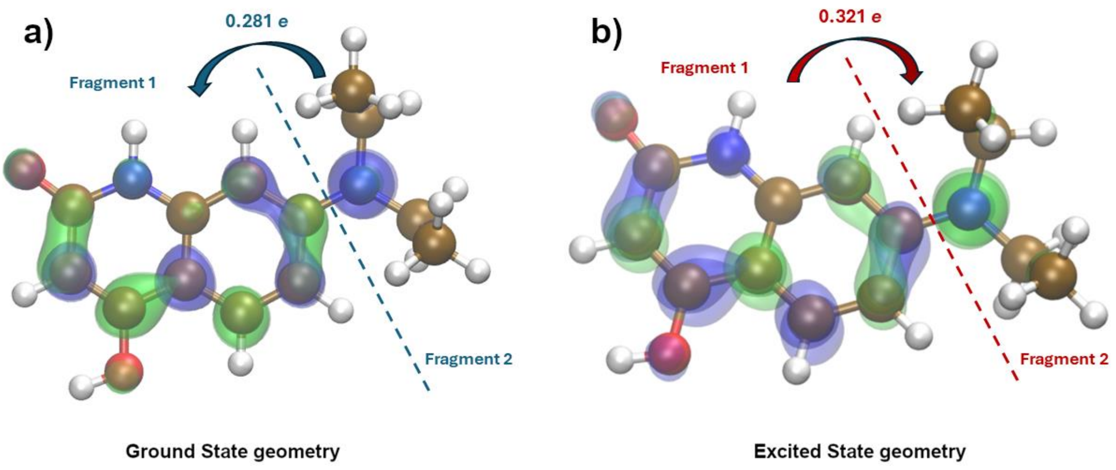

Figure S8. Electron(blue)-hole(green) distributions of the first electronic excited state of aqueous **QD** at either the ground state (a) or excited state (b) optimized geometry at C-PCM/B3LYP(D3)/6-311++G** level of computation.

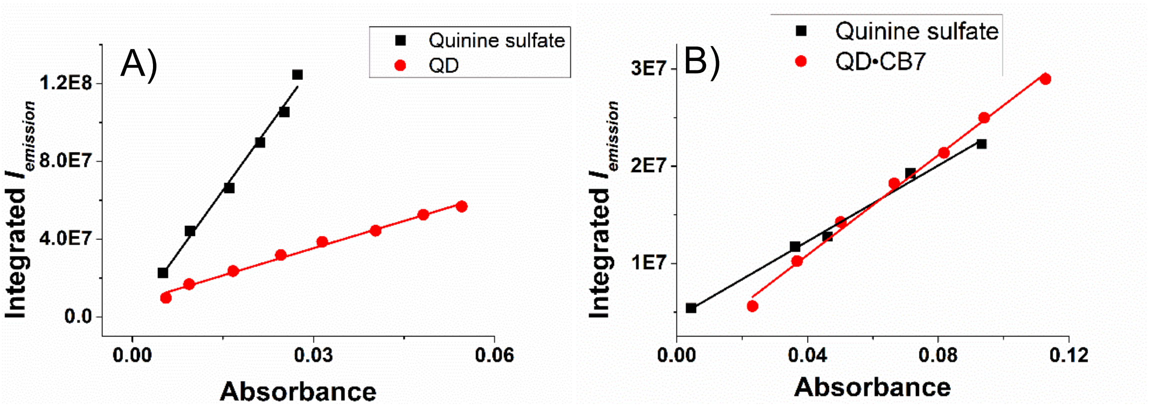

Figure S9. Integrated fluorescence intensity *vs* absorbance for quinine sulfate (black dots) and A) quinolinone derivative (red dots) B) complex **QD•CB7** (red dots) used for the determination of quantum yields.

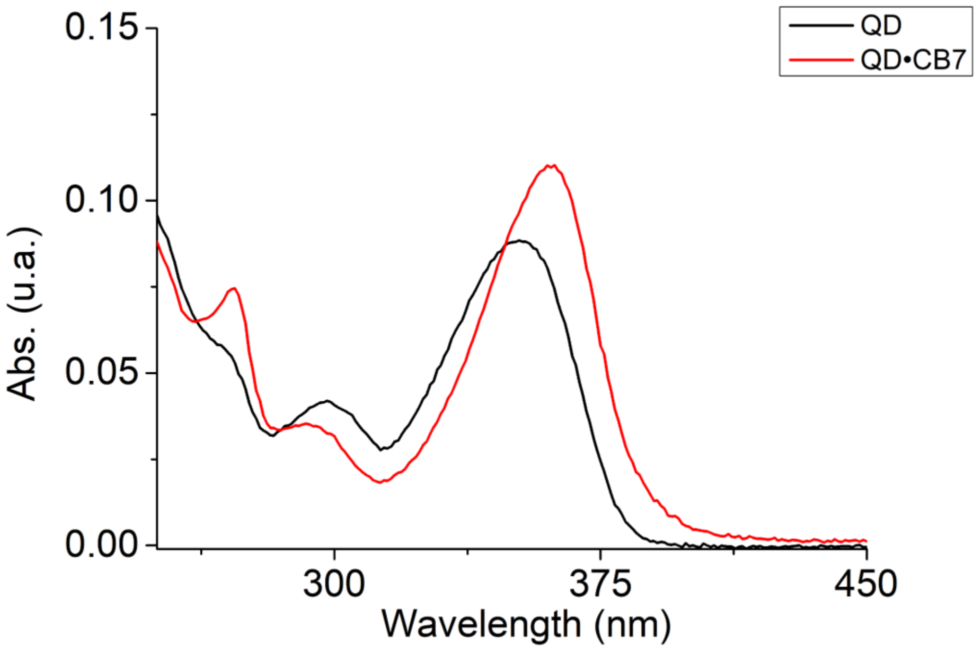

Figure S10. Absorbance for **QD** (black line) and **QD•CB7** (red line) supramolecular complex in water/DMSO mixture (99/1) at room temperature, respectively.

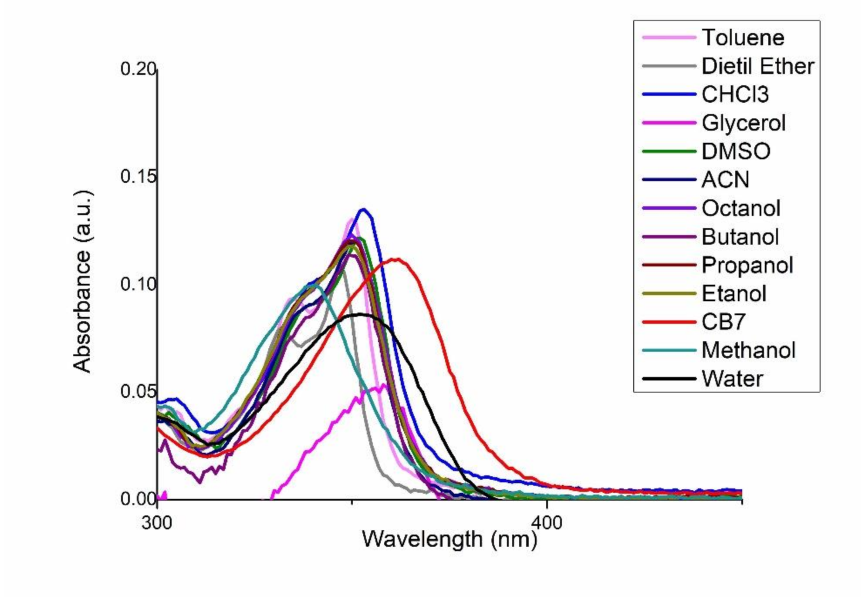

Figure S11. UV-Vis spectra of **QD** in solvents with varying polarities at room temperature.

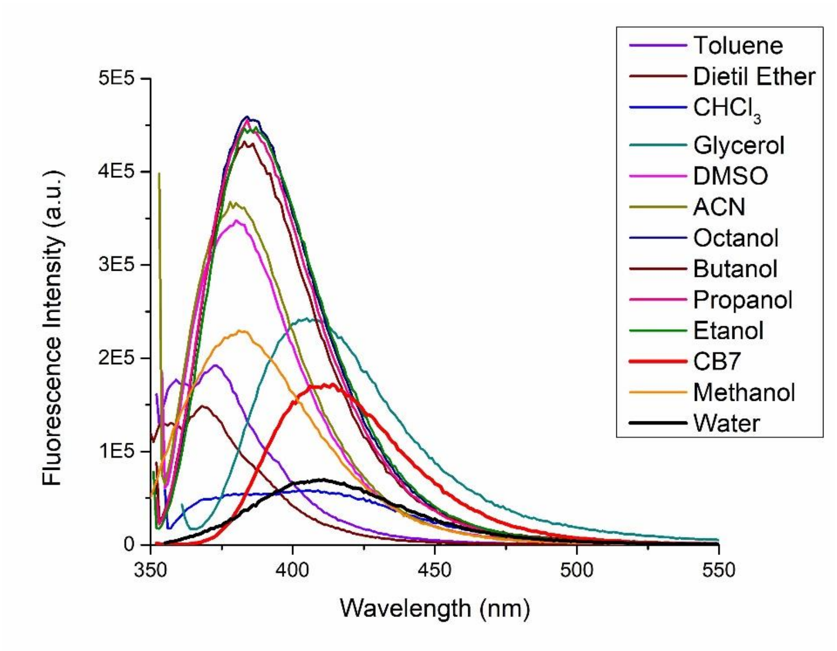

Figure S12. Emission spectra of **QD** in solvents with varying polarities at room temperature.

Table S4. Properties of solvent used and their effect on photophysical properties of **QD**.

**Solvent**	Viscosity (cP)	$E_t^{n(1)}$	l_{max}^{abs} (nm)	l_{max}^{emi} (nm)	t_1 ns / (A1)	t_2 ns / (A2)
**Toluene**	0.59	0.099	350	373	0.03 (99)	0.39 (1)
**Diethyl Ether**	0.24	0.117	347	368	0.13 (99)	1.42 (1)
**Chloroform**	0.56	0.259	353	409	0.65 (54)	4.2 (46)
**Glycerol**	1412	0.400	357	405	0.44 (30)	3.06 (70)
**Dimethyl Sulfoxide**	1.99	0.444	352	381	0.16 (72)	1.97 (18)
**Acetonitrile**	0.35	0.460	351	381	0.06 (96)	2.20 (4)
**Octan-1-ol**	7.5	0.537	350	385	0.08 (56)	2.74 (44)
**Butan-1-ol**	2.98	0.586	350	384	0.09 (69)	2.99 (31)
**Propan-1-ol**	2.2	0.617	350	385	0.18 (33)	2.75 (67)
**Ethanol**	1.2	0.654	350	385	0.03 (84)	2.82 (16)

**CB7**	-	-	361	410	0.15 (1)	4.87 (99)
**Methanol**	0.59	0.762	340	381	0.06 (98)	3.02 (2)
**Water**	1	1.000	351	381	0.59 (96)	1.39 (4)

(1) These values were used previously in https://doi.org/10.1021/acsphyschemau.5c00083
